# Strawberry: Fast and accurate genome-guided transcript reconstruction and quantification from RNA-Seq

**DOI:** 10.1371/journal.pcbi.1005851

**Published:** 2017-11-27

**Authors:** Ruolin Liu, Julie Dickerson

**Affiliations:** Department of Electrical and Computational Engineering, Iowa State University, Ames, Iowa, United States of America; University of Pennsylvania, UNITED STATES

## Abstract

We propose a novel method and software tool, Strawberry, for transcript reconstruction and quantification from RNA-Seq data under the guidance of genome alignment and independent of gene annotation. Strawberry consists of two modules: assembly and quantification. The novelty of Strawberry is that the two modules use different optimization frameworks but utilize the same data graph structure, which allows a highly efficient, expandable and accurate algorithm for dealing large data. The assembly module parses aligned reads into splicing graphs, and uses network flow algorithms to select the most likely transcripts. The quantification module uses a latent class model to assign read counts from the nodes of splicing graphs to transcripts. Strawberry simultaneously estimates the transcript abundances and corrects for sequencing bias through an EM algorithm. Based on simulations, Strawberry outperforms Cufflinks and StringTie in terms of both assembly and quantification accuracies. Under the evaluation of a real data set, the estimated transcript expression by Strawberry has the highest correlation with Nanostring probe counts, an independent experiment measure for transcript expression. Availability: Strawberry is written in C++14, and is available as open source software at https://github.com/ruolin/strawberry under the MIT license.

## Author summary

Transcript assembly and quantification are important bioinformatics applications of RNA-Seq. The difficulty of solving these problem arises from the ambiguity of reads assignment to isoforms uniquely. This challenge is twofold: statistically, it requires a high-dimensional mixture model, and computationally, it needs to process datasets that commonly consist of tens of millions of reads. Existing algorithms either use very complex models that are too slow or assume no models, rather heuristic, and thus less accurate. Strawberry seeks to achieve a great balance between the model complexity and speed. Strawberry effectively leverages a graph-based algorithm to utilize all possible information from pair-end reads and, to our knowledge, is the first to apply a flow network algorithm on the constrained assembly problem. We are also the first to formulate the quantification problem in a latent class model. All of these features not only lead to a more flexible and complex quantification model but also yield software that is easier to maintain and extend. In this method paper, we have shown that the Strawberry method is novel, accurate, fast and scalable using both simulated data and real data.

This is a *PLOS Computational Biology* Methods paper.

## Introduction

Transcript-level quantification is a key step for detecting differential alternative splicing and differential gene expression. A number of computational methods have been developed for estimation of transcript abundances [1–9]. However, many of the methods [4–9] rely on existing gene annotations and limits the use of such methods because even for the model organisms like *Drosophila melanogaster* new isoforms are discovered all the time under different tissues and/or conditions (*Pachter*, *2011*, *Models for transcript quantification from RNA-Seq*). In addition, Liu et al. has shown that incomplete annotation is a major factor that negatively affects quantification accuracy for detecting alternative splicing [10]. Thus, transcript-level quantification should be coupled with transcript assembly when dealing with RNA-Seq data. Pure de novo assembly of raw RNA-Seq is very challenging. Genome-guided methods, instead, assemble aligned RNA-Seq reads into transcripts, taking advantage of (if possible) a finished and high quality genome assembly and the-state-of-art spliced alignment algorithms.

Two strategies have evolved for tackling transcript assembly and quantification after RNA-Seq reads have been aligned to reference genome: simultaneous transcript construction and expression quantification vs. sequential transcript construction then expression quantification. Clearly, transcript reconstruction and quantification are closely related and many methods try to solve both simultaneously [3, 11–13]. These methods usually exhaustively enumerate all possible transcripts and then use regularization to get rid of unlikely transcripts when calculating their expression. The *L*1 penalty is commonly used to favor sparse transcript solutions [13]. Another strategy involves breaking the problem up in a step-by-step manner, like Cufflinks. First, reconstruct a set of transcripts, and then performs quantification on the transcripts. The latter is a more conservative strategy and usually leads to “maximum precision vs. maximum sensitivity” [14] compared to the former.

### Method overview

**Fig 1:**
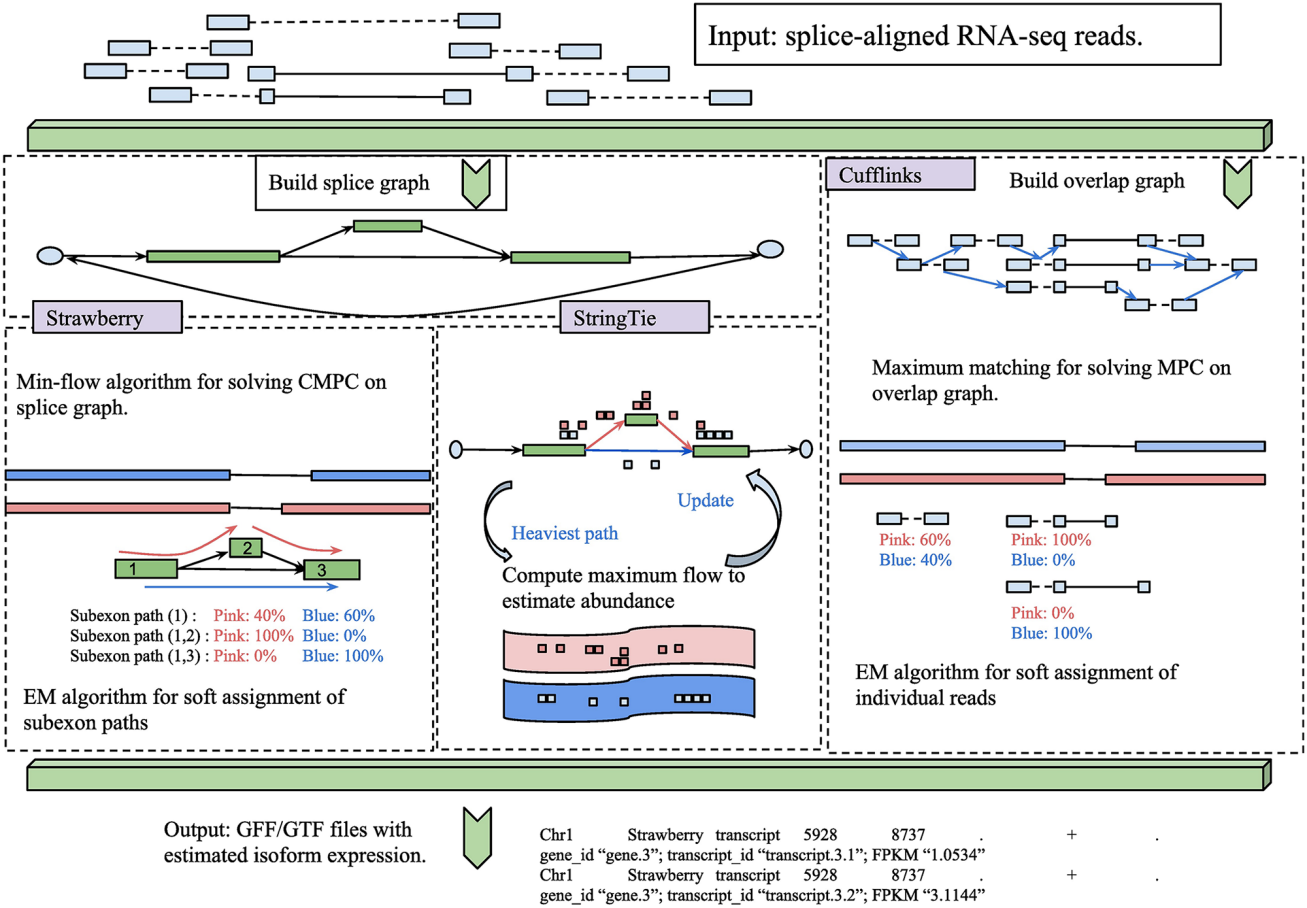
Overview of the algorithm of Strawberry, compared to StringTie and Cufflinks. All methods begin with a set of RNA-Seq alignments and output transcript structures and abundances in GFF/GTF format. Strawberry uses a min-flow algorithm for solving Constrained Minimum Path Cover(CMPC) problem on splicing graph, followed by assigning subexon paths to compatible assembled transcripts. In quantification step, all of the RNA-Seq read alignments on each subexon path as a whole are the subject of the EM algorithm.

Strawberry consists of two modules: assembly module and quantification module. The two modules work in a sequential manner (Fig 1). Strawberry is a genome-guided transcript-level assembler and quantification tool. It takes aligned RNA-Seq data in BAM format and output a gene annotation file in gff format with estimated transcript abundances. Using alignment format as input allows Strawberry to take advantages of the latest reference genome (if possible, a finished and high-quality one) and stat-of-the-art splice-awareness aligners. Strawberry is designed for Illumina pair-end reads. To be clear in this article, a read-pair refers to aligned paired-end reads with sequences observed at both ends and unknown sequence in between and a read refers to either the upstream or downstream observed sequence of a read-pair. For single-end reads, replace the terminology “read-pair” with “read” and proceed.

The assembly module of Strawberry seeks a parsimonious representation of transcripts which best explains the observed read-pairs with the aid of flow network algorithms. The read-pairs are converted to splicing graphs where the nodes are subexons and edges are splice alignments. FlipFlop [3], StringTie [2] and Traph [11] also use network flow algorithm, but for different purposes. StringTie and Traph renounce the likelihood-based approach and solve transcript assembly and quantification as optimization problems and solve the two problems simultaneously in a flow network framework build upon on splice graph. The difference is that Traph uses a min-flow algorithm to find a set of flows that minimize the difference between the flows and the observed coverages, while StringTie uses an iterative algorithm to harvest the heaviest path and then uses maximum flow to estimate their expression. Here, a flow can be understood as a transcript with uniform coverage along it. Although also using flow network, FlipFlop constructs a penalized likelihood model. The penalized likelihood model is carefully designed to be convex and the estimation problem can be cast into a convex-cost min-flow. Different from all of them, Strawberry uses a min-cost circulation flow to solve a parsimonious assembly problem. If the underlying sequence of a read-pair contains an unsequenced portion, such as the insert, this read-pair might indicate necessary paths that are usually neglected by other methods [15], while Strawberry explicitly converts them to graph constraints. In a nutshell, StringTie uses a flow network to calculate transcript expression; Traph and FlipFlop use flow networks to concurrently solve transcript identification and quantification. Strawberry is the only one that applies a flow network to an assembly problem. The assembly problem that Strawberry is solving is also unique. It is a constrained assembly problem that is tailored for paired-end reads by converting them to graph path constraints (see method section).

The quantification model of Strawberry is based on a latent class model with an effective data collapsing mechanism, which utilizes the same graph topology used in assembly to reduce the individual reads to subexon path counts. A subexon is a maximal portion of covered region (covered by reads) without any splice junctions. And subexon path is regarded a set of ordered subexons. The subexon path representation allows Strawberry to save computational cost and model nonuniform reads distribution along transcripts. To the best of our knowledge, the concept of subexon path was first proposed in [6]. However, it can be seen as a modification/extension of the idea of *maximum collapsing* in [16]. Although using same data collapsing mechanism, Rossell et al. uses a Bayesian framework and does not have a joint estimation of transcript proportion and coverage bias effect [6]. While Strawberry applies a conditional multinomial distribution for the subexon paths and estimates the transcript proportion and coverage effect simultaneously in the mixture model. The change from a non-parametric model in [6] to a multinomial model in Strawberry permits better model expandability.

Strawberry is designed to be versatile and modular. It is possible to skip the assembly step and just run quantification module against an external set of transcripts, e.g. those from gene annotations. In this case, Strawberry reduces any overlapping set of isoforms to a splicing graph consisting of subexons and subexon paths. The external set of transcripts can also be used by Strawberry to help with assembly. Finally, Strawberry reports the calculated transcript expression in the units of FPKM (Fragments Per Kilobase of transcript per Million mapped reads) and TPM (Transcripts Per Kilobase Million).

## Results

### Ground truth simulated data and programs to compare

We compare Strawberry to two state-of-the-art programs, Cufflinks v2.2.1 [1] and StringTie v1.3.3 [2], on three simulated data sets, *RD25* *RD60* and *RD100*. The only difference among these three data sets is the average sequencing depth. Roughly speaking, RD25 contains ∼2.5 million, RD60 ∼6 million and RD100 ∼10 million reads. These data were generated by the procedure used in [10]—100bp paired-end reads generated from 5800 multi-isoform Arabidopsis genes on genome version TAIR10 [17] using Flux Simulator [18]. This simulation was repeated 10 times so that each data set consists of 10 RNA-Seq libraries. Those simulated reads were then mapped onto the Arabidopsis TAIR10 genome assembly using Tophat2 [19] and HISAT2 [20]. Since plant genomes have shorter introns than mammals, all the programs ran on the default parameters except for the maximum intron length, which was set to 5000 bp.

To evaluate Strawberry’s performance on higher eukaryotes, we also compare the three programs using simulated human RNA-seq data. To avoid possible simulation bias, we choose a different simulator called Polyester [21]. Polyester requires a count matrix, where each row represents a transcript and each column contains the read counts for a sample, as an input. To generate this count matrix, we downloaded 6 samples from the GEUVADIS data base [22] and aligned them with HISAT2. Then Cufflinks was used to estimate transcript expression. All transcripts were selected from loci which have at least two isoforms with FPKM >1.0 for all six samples. This human simulation is referred to as *GEU*. Compared to *RD100*, *GEU* has relatively longer read length (150 bp paired-end) and longer fragment length (700 bp in average). This read length and fragment size are intended for the latest illumina sequencer NextSeq.

### Comparing assembly accuracy

We use a Cufflinks module called Cuffcompare http://cole-trapnell-lab.github.io/cufflinks/cuffcompare/index.html to compare the assembled transcripts or transfrags to the reference transcripts since the reads are all simulated based on the reference transcripts. We use Cuffcompare’s evaluation algorithm which implements typical gene finding measures of recall and precision [23]. For example, the recall of an exon is the percentage of number of corrected exons divided by the number of actual exons and precision is the number of correct exons divided by the number of predicted exons. Determination of transcription start and end sites is a known weakness of RNA-Seq and impairs its application on identification of transcript boundaries [24]. Thus, Cuffcompare defines a correct transcript as the chain of introns that match with the reference, leaving possible variances in the first and last exon.

**Fig 2:**
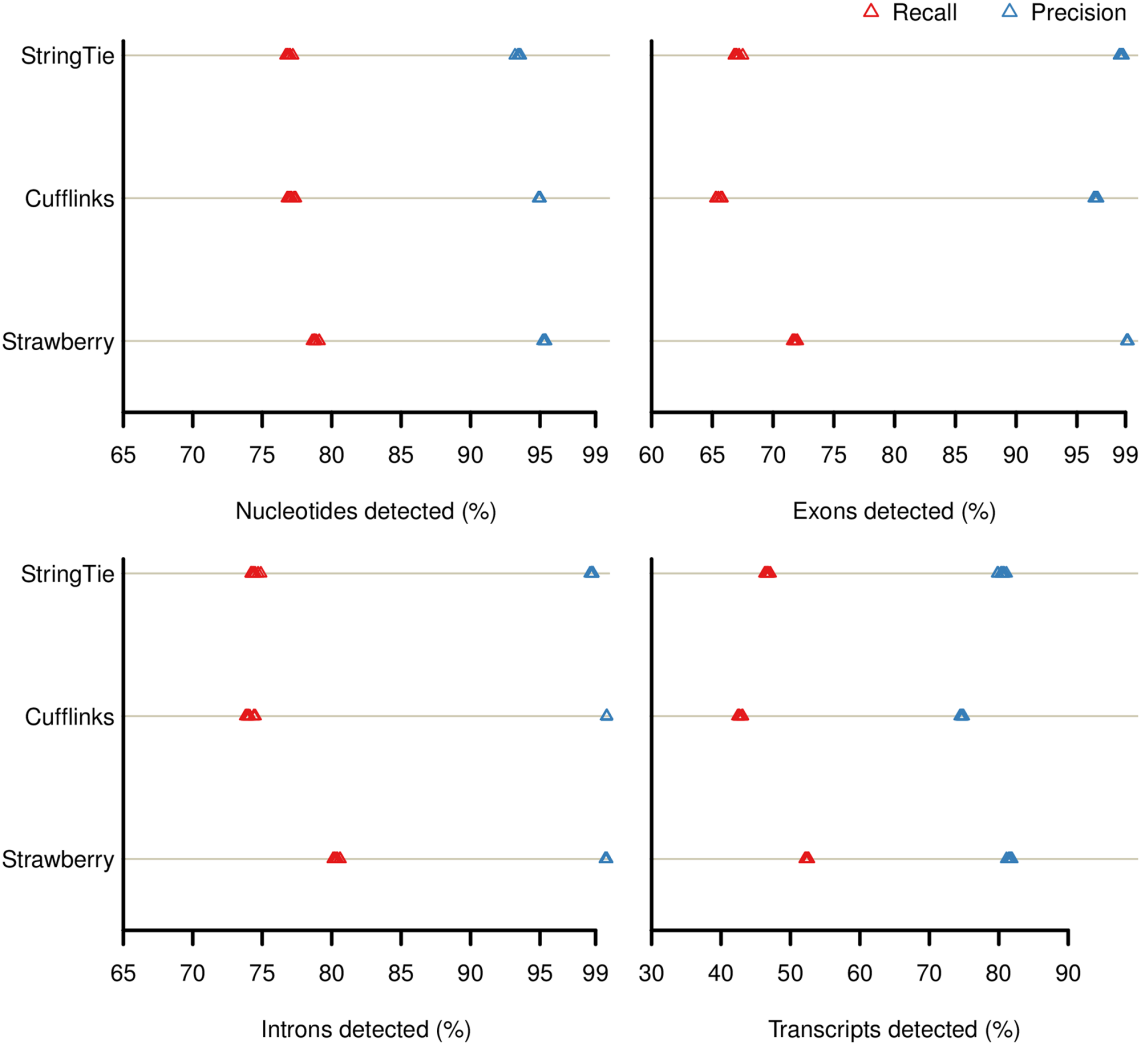
Recall and precision at the nucleotide, exon, intron and transcript level. StringTie, Cufflinks and Strawberry were run on data *RD100*, which is a simulated Arabidopsis RNA-Seq data set.

We first assessed the genome-guided assembly accuracy of the three programs using simulated Arabidopsis data set. The degree to which transcripts reported by each method matched the reference annotation at the nucleotide, exon, intron and transcript level for three different sequencing depths are shown in (Fig 2, S1 and S2 Figs). In all comparisons, Strawberry has higher recall as well as precision. In *RD100* data, for example, Strawberry averages 71.78%, 80.36%, 52.35% on recall at exons, intron, and full transcripts level respectively, followed StringTie, 67.03%, 74.41%, 46.65% and then Cufflinks, 65.51%, 74.09%, 42.76%. For all the methods, the recall decreases as sequencing depth decreases while the precision remains at a high level and doesn’t change much. This indicates that although lower read depths make it harder for these methods to recover the true signal, the results are still very reliable. Correct detection of full transcripts using RNA-Seq data is still a very challenging task for all assemblers. Given sufficient sequencing depth (*RD100*), all methods can correctly retrieve more than 65% exons, and 75% intron but only around 50% of the full transcripts. On the other hand, precision for exons and intron detection are very high for all methods, averaging 98–99%. For transcript detection, Strawberry’s average precision is 81.62%, while StringTie is at 80.46% and Cufflinks at 74.68%. For the methods that parsimoniously assemble reads into transcripts, this may indicate some room for improvement—although the individual exons and introns are correctly recovered, the ways to stitch them together are still not optimal. We further conducted a paired t-test to evaluate the statistical significance of the difference in F1 score (the harmonic mean of recall and precision) between Strawberry and the other tools (p value = 7.02e-12 when compared to StringTie, and p value = 1.947e-14 when compared to Cufflinks).

**Fig 3:**
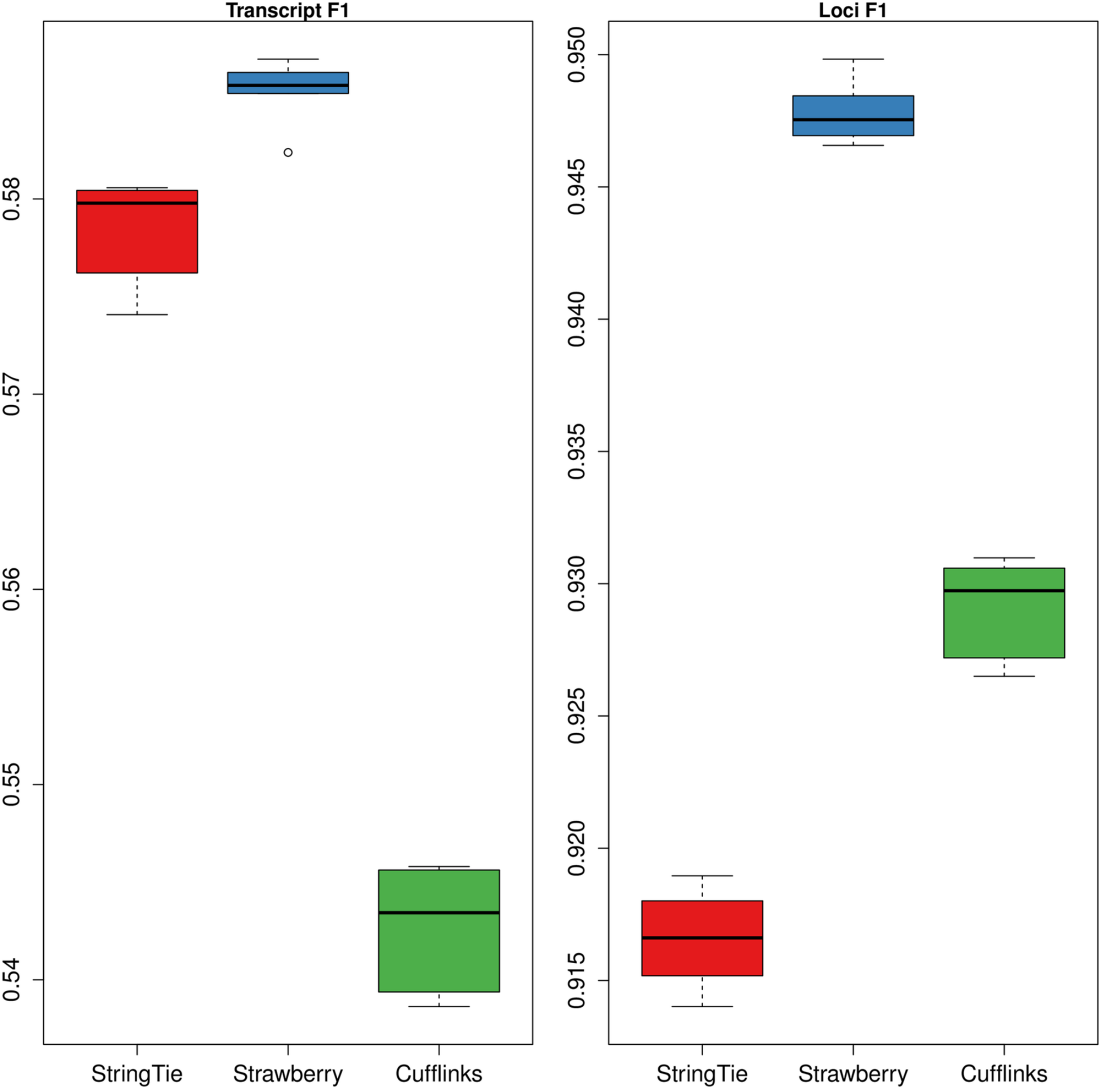
Box plots of F1 scores at the transcript and loci level. StringTie, Cufflinks and Strawberry were evaluated on data *GEU*, which is a simulated Human RNA-Seq data set.

Next, we evaluated the methods using *GEU*. Overall, we observe that the F1 values at transcript level are roughly at the same level as in *RD100*, and Strawberry clearly maintains its lead, followed by StringTie (Fig 3). However, the gap between Strawberry and StringTie is smaller compared to *RD100*. Again, a paired t-test of F1 scores is used, yielding p value = 5.614e-03 when compared to StringTie, and p value = 2.965e-09. Strawberry also achieves the best F1 score at gene level (Fig 3), and Cufflinks performs better than StringTie at gene level. When it comes to exon and intron levels comparison, however, StringTie clearly performs better than Strawberry and Cufflinks, see S5 Fig. This suggests Strawberry still has room to improve the detection on exon and intron level for human, which can lead to higher transcript reconstruction rate.

### Comparing quantification accuracy

Let *x*_*i*_ be the true value of the FPKM for transcript *i* based on ground truth simulated data and *y*_*i*_ be the predicted FPKM. If log transformation is taking, these FPKM values were incremented by 1 before log transformation to avoid infinite numbers. We adopt the metrics defined in *Patro et*.*al 2017* [4].

1. Proportionality correlation
  $$\begin{array}{c} \rho_{p}=\frac{2\text{Cov}\{\log x,\log y\}}{\text{Var}\{\log x\}+\text{Var}\{\log y\}} \end{array}$$ (1)
2. Spearman correlation of between the true FPKM values and predicted FPKM values.
3. Mean Absolute Relative Difference (MARD), which is computed using the absolute relative difference ARD_*i*_ for each transcript *i*:
  $$\begin{array}{c} {\text{ARD}}_{i}=\frac{|x_{i}-y_{i}|}{0.5|x_{i}+y_{i}|}, \end{array}$$ (2)
  MARD is the mean value of the {ARD_*i*_|*i* ∈ 1, …, *I*}. ARD is bounded above by 2 and below by 0 and smaller value indicates a better prediction. *Patro et al*. [4] computes MARD on the number of reads deriving from each transcript which is commensurable to FPKM values.

**Fig 4:**
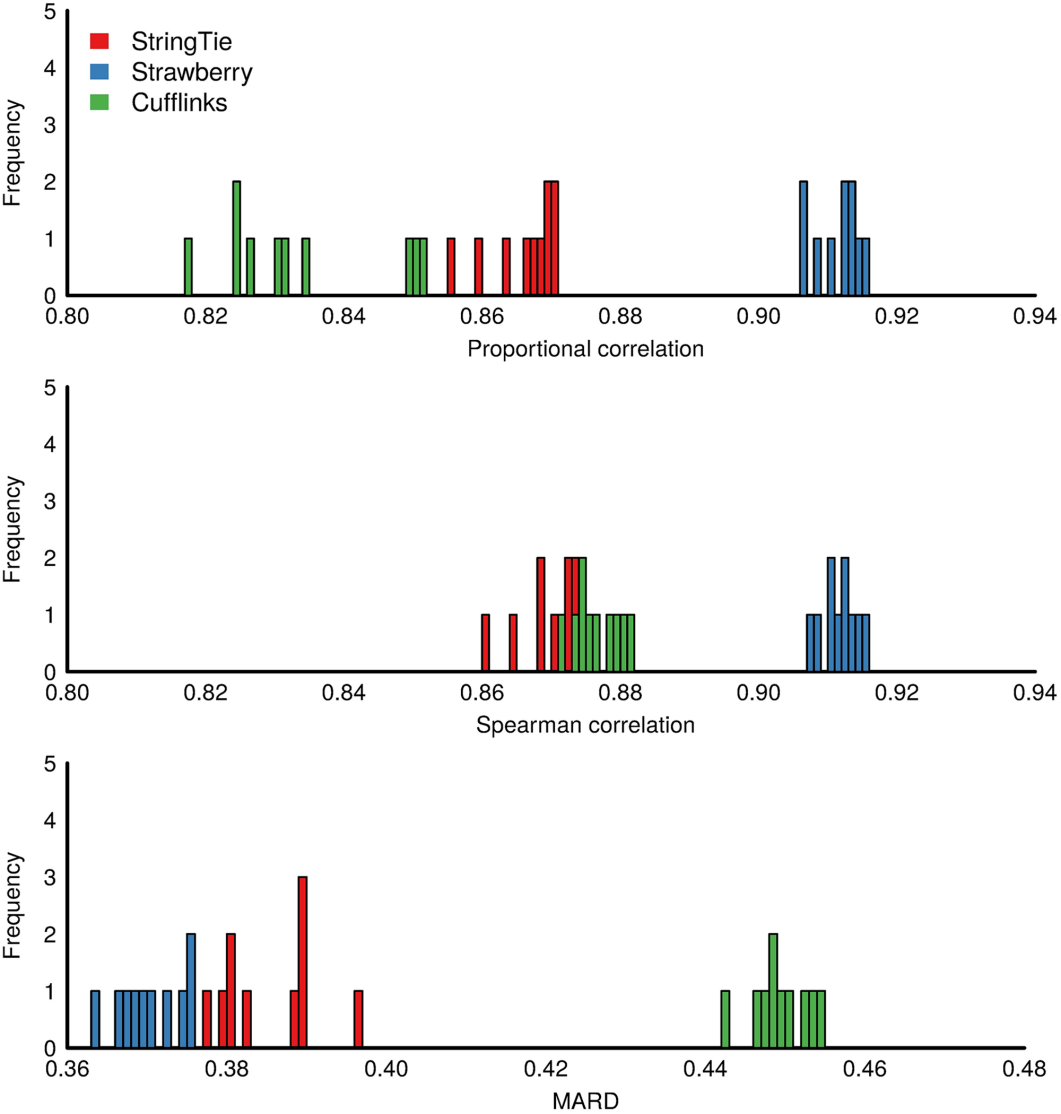
Frequency plot of Proportional correlation, Spearman correlation, Mean Absolute Relative Difference (MARD) for the 10 replicates in *RD100*, which is a simulated Arabidopsis data.

Again, we first evaluate the methods using simulated Arabidopsis data. Fig 4, S3 and S4 Figs show the histogram of the three measures over 10 replicates for all three read depth data sets *RD100*, *RD60* and *RD25* respectively. In these simulations, It can be seen that these methods are all well separated in terms of the all evaluation metrics except for only one case in which StringTie and Cufflinks are virtually tied over Spearman correlation in *RD60* data (S3 Fig). In the case of *RD100* data, Strawberry averaged 0.911, 0.912, and 0.370 on Proportional correlation, Spearman correlation and MARD respectively, followed by StringTie, 0.866, 0.869, 0.385 and then Cufflinks, 0.834, 0.876, 0.450. Cufflinks outperforms StringTie in terms of Spearman correlation but not the other two metrics. Like the assembly results, the sequencing depth seems to have a uniform impact on the quantification accuracy and all methods favor the highest read depth. It is worth mentioning that our enumeration of read depths only focuses on down sampling. Overall, Strawberry outperforms the other methods under all evaluation metrics and sequencing depth and StringTie performs better than Cufflinks. However, the distance between the second and third place is less than the distance between the first and second place. We also observe that Strawberry and StringTie have less variability in results than Cufflinks did, suggesting they are more consistent in terms of their estimates.

**Fig 5:**
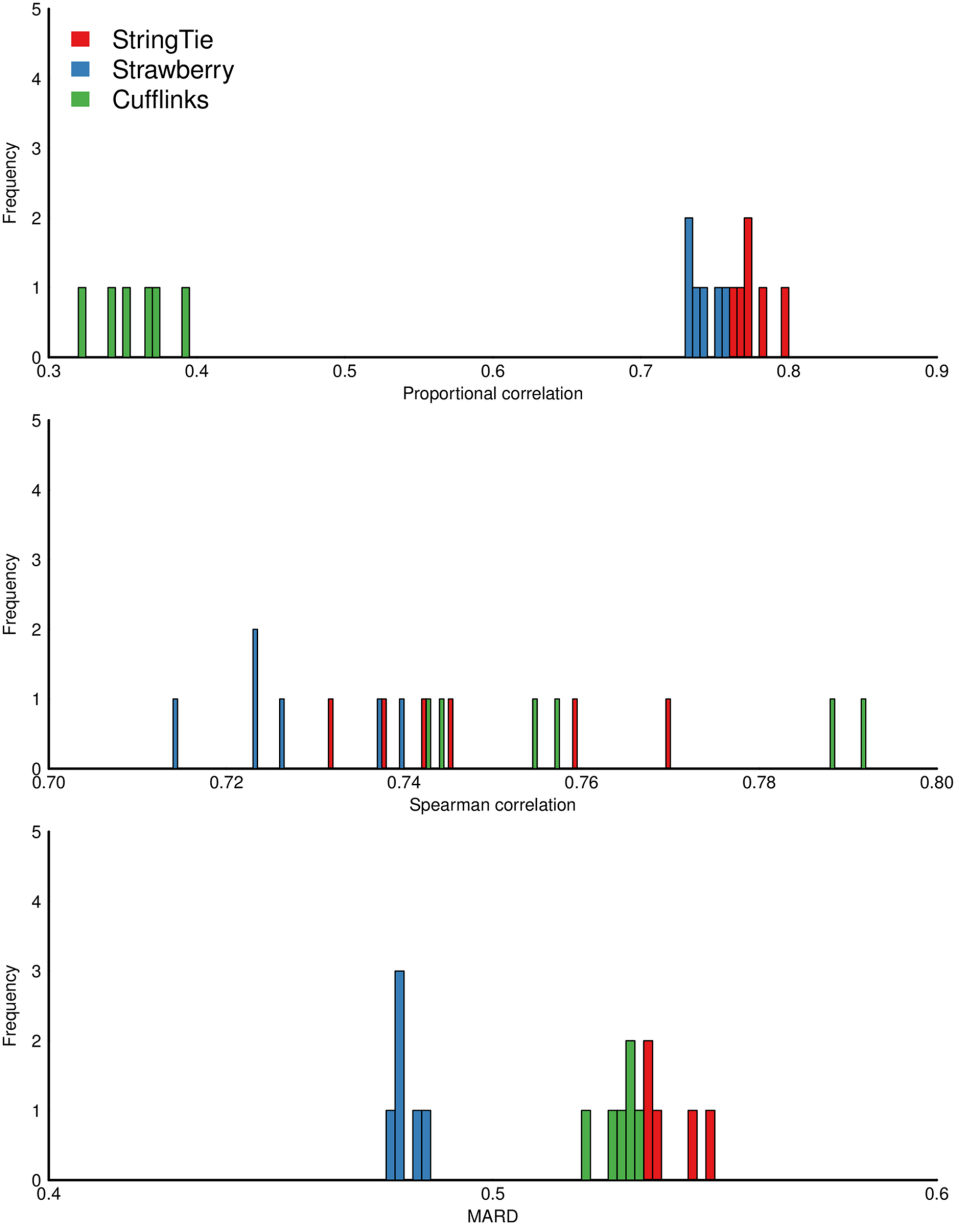
Frequency plot of Proportional correlation, Spearman correlation, Mean Absolute Relative Difference (MARD) for the 6 samples in *GEU*, which is a simulated Human data. These statistics are calculated based on the predicted FPKM values of all reconstructed transcripts and the true FPKM values used in the simulation.

When evaluated on simulated human RNA-Seq data, all three methods have lower correlations and higher relative differences compared with the true FPKM values. The order of the methods’ performances slightly changes based on different evaluation metrics (Fig 5). Strawberry has the lowest average MARD across the 6 samples compared to StringTie and Cufflinks (Table 1). When the methods are compared using Spearman correlation, the differences among the three methods are the smallest. Cufflinks performs poorly under proportionality correlation (averaged at 0.3573). StringTie achieves the highest average proportionality while Strawberry is the second. Fig 5 compares the FPKM value of each predicted transcript against its best possible matched known transcript’s true FPKM value. S6 Fig removes the predicted transcripts that are partially matched and only keeps the transcripts that fully match the known transcripts, i.e., class code equal to “=” in the Cuffcompare result. In this “match only” case, all statistics improved significantly for all the methods, and Strawberry performs the best in every comparisons (Table 1).

**Table 1:** Averaged Spearman correlation, Proportional correlation, Mean Absolute Relative Difference (MARD) for the 6 samples in *GEU*, which is a simulated Human data. These statistics are calculated based on the predicted FPKM values of 1) all reconstructed transcripts 2) only transcripts that match the known, and the true FPKM values used in the simulation.

	Method	Avg. Sp.	Avg. Prop.	Avg. MARD
All transcripts	Strawberry	0.7272	0.7430	0.4801
	StringTie	0.7476	0.7759	0.5392
	Cufflinks	0.7631	0.3573	0.5287
Match only	Strawberry	0.8706	0.8706	0.3144
	StringTie	0.8517	0.8704	0.4068
	Cufflinks	0.8614	0.6621	0.4561

### Real RNA-Seq data

To demonstrate Strawberry utility on real data, we tested all three programs on the Homo sapiens HepG2 data from *Steijger et al*. [24]. The data was downloaded from http://www.ebi.ac.uk/arrayexpress/experiments/E-MTAB-1730/, which includes alignment results from a library of 100 million 76bp paired-end Homo sapiens RNA-Seq reads and a total of 140 NanoString probe counts. These 140 probes targeted 109 genes, designed against specific transcripts. NanoString counts were then compared to the highest FPKM value reported for transcripts consistent with the probe design [24]. We followed the same procedure used in *Steijger et al*. except for using the Tophat2 alignment result and Cuffcompare for finding the best matching transcripts. Correlations between the log-transformed FPKM reported by each method and NanoString count was calculated. Strawberry again is clearly the front-runner, correlation increased by 10.3%, 5.26% compared to Cufflinks and StringTie respectively (Table 2). The number of probes having matched transcripts were very close for all three methods.

**Table 2:** Correlation of FPKMs and probe counts on real RNA-Seq data HepG2. NanoString counts were compared to the FPKM values reported for three programs. The number of probes which have matching transcripts is reported on the last line.

	Strawberry	Cufflinks	StringTie
Spearman Corr.	0.640	0.580	0.608
Num. of matches	82	82	83

It’s worth mentioning that the numbers reported here may not be directly comparable to the numbers in *Steijger et al*. because we use a different aligner. In *Steijger et al*., STAR [25] was used as the default aligner. However the STAR alignment result, as a supplementary file in their paper, does not contain *XS*, which is used in the BAM format to suggest the transcription orientation from splice site dinucleotides, such as GT-AG.

**Fig 6:**
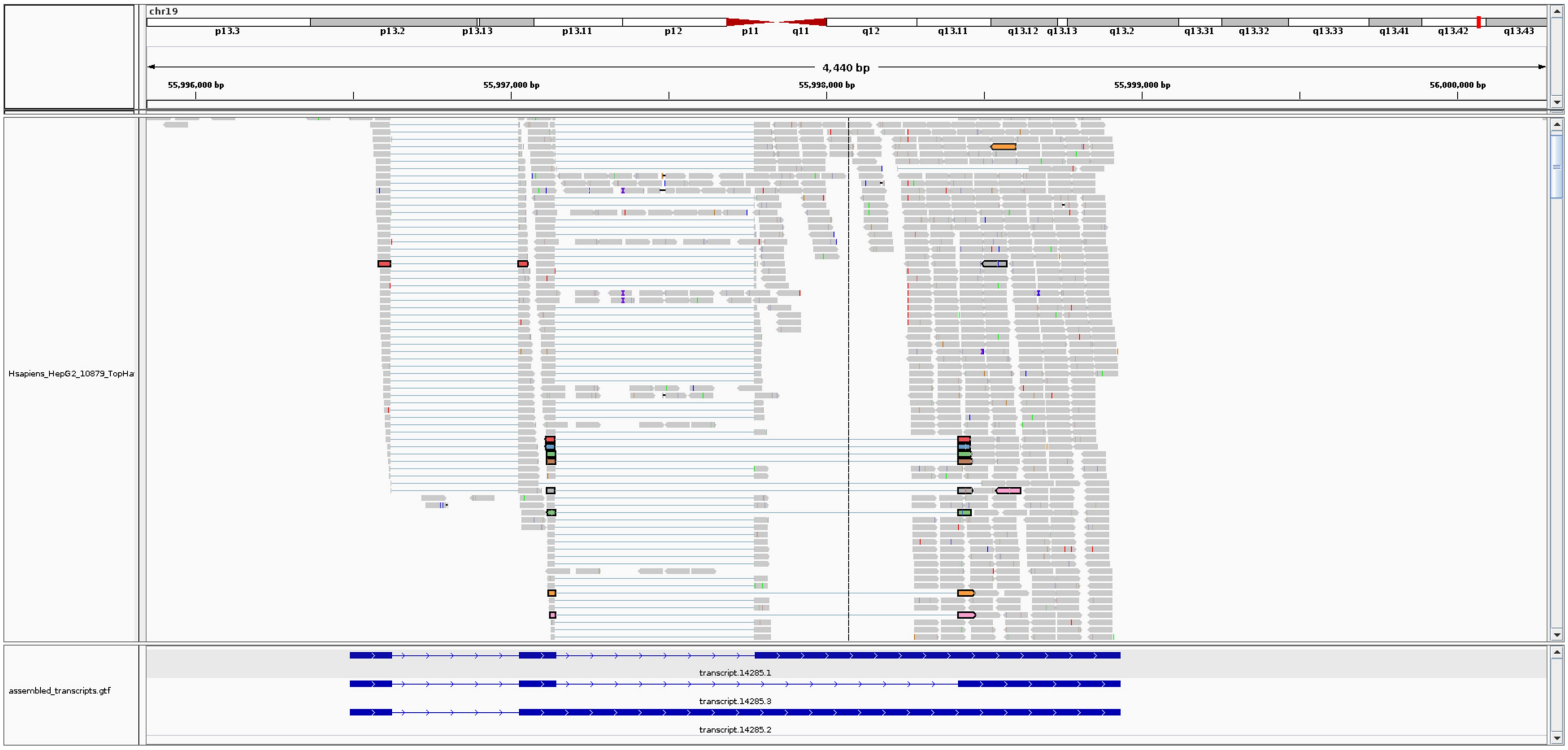
Read alignments and reconstructed transcripts at gene NAT14 using HepG2 data. A new isoform, transcript.14285.3 (shown as the middle one), has been identified by Strawberry. The junction reads that support the new AS event (alternative 3 splice site) are highlighted. The two ends of a read-pair are in the same color. A total 7 uniquely mapped read-pairs supports the novel junction. This figure is made by IGV (http://software.broadinstitute.org/software/igv/).

Fig 6 shows an example of a novel isoform discovered by Strawberry in the HepG2 data. At locus ENSG0000009097, Strawberry reconstructs three isoforms. Two of them matches known isoforms ENST00000591590 and ENST00000205194 based on GRCh37 Ensemble annotation. The third isoform, transcript.14285.3, contains a novel splicing junction which is supported by 7 uniquely aligned read-pairs. Strawberry predicts the new isoform at a fraction of 0.277 in all the three predicted isoforms.

### Running time

**Fig 7:**
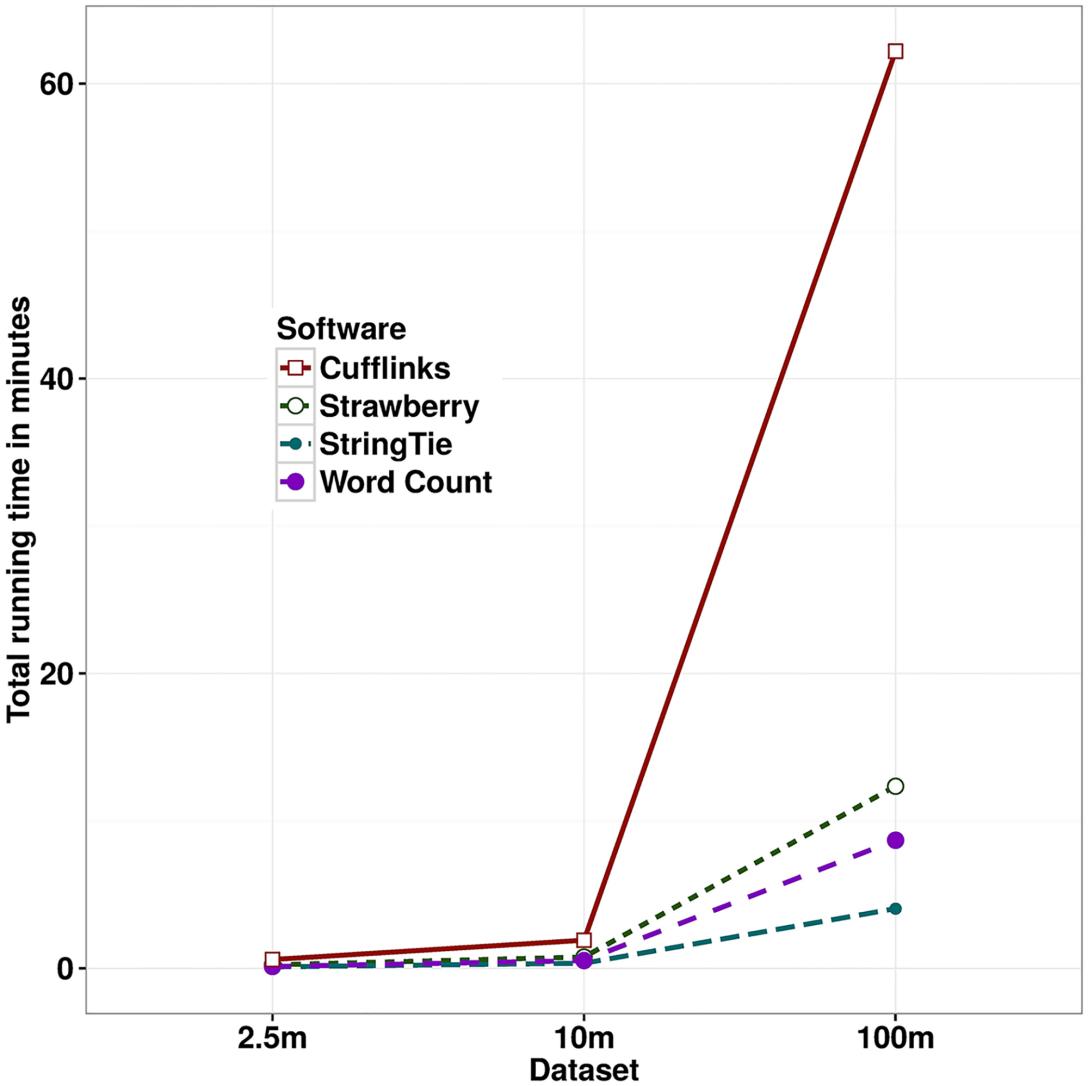
Running time in minutes of Cufflinks, Strawberry, linux word count and StringTie(ordered by slowest to fastest) on textitRD25(2.5 million reads), *RD100*(10 millions reads), and *HepG2* data(100 millions reads).

The running time of all three program plus a simple linux word count program on RD25, RD100, and HepG2 are plotted in Fig 7. For the HepG2 data, Cufflinks tooks 62.2 min, Strawberry 12.35 min and StringTie 4.05 min. All programs were run using 8 threads on a Dell Precision T1650, equipped with Intel Core i7-3770 CPU and 16 GB RAM. Each program was given the aligned data in BAM format and the time spent on alignment is not included. To see how well these programs scale when input grows in size, we ran a simple single thread linux word count program *wc* (which is known to have linear complexity) on the SAM format of the same data. Surprisingly, StringTie is even faster than *wc*(which uses 8.69 min), and it demonstrates the simplicity of StringTie algorithm. Strawberry also scales well compared to *wc*. Cufflinks running time shoots up when the number of RNA-Seq reads grows to 100 million. Cufflinks and Strawberry both use the EM algorithm for assigning ambiguous reads to transcripts. The EM algorithm is a time consuming algorithm but the reduced data representation used in Strawberry makes it almost 5 times faster than Cufflinks.

## Discussion

Strawberry adopts a step-by-step approach for transcript assembly and quantification of expression levels. We believe it is critical to assemble the transcriptome before carrying out quantification since every eukaryotic RNA-Seq experiment is likely to generate unknown transcripts even for the well-annotated species. Our previous study of alternative splicing has shown that an incomplete genome annotation can have a huge negative impact on the detection accuracy of alternative splicing events [10]. Strawberry avoids strictly using gene annotations for quantification and is able to assemble novel isoforms. However, with high-quality annotation, Strawberry can take advantage of the annotation and yield a better assembly result. The genome guided assembly is enabled by “-g” option.

Strawberry’s transcriptome assembly takes advantage of the latest genome assembly and state-of-art splice-awareness aligners and is usually more accurate than the de novo assemblers. However, this makes Strawberry reliant on alignment results. Another limitation of current Strawberry’s assembly is the lack of detection of alternative promoter usage and alternative polyadenylation. Unlike other alternative splicing events, de-novo detection of alternative promoter usage and alternative polyadenylation can not be inferred from junction alignments and requires some sophisticated read depth models because of the intrinsic noisiness around transcription start and end sites introduced by RNA-Seq.

Compared to current approaches such as FlipFlop, Strawberry’s assemble-then-quantify procedure cannot best utilize the quantification information in the assembly step. This is because for short-read technologies, such as Illumina, the local estimates of relative abundance are the only information available for phasing distant exons during assembly. However, Strawberry’s flow network algorithm is able alleviate this phasing problem by converting the exon and junction coverage into the weighs of the flows. As a result, for example, the exons and exon-exon junctions which have similar coverages will tend to form one path by the optimization algorithm.

Both Cufflinks and Strawberry use the EM algorithm for optimizing the likelihood functions. However, because of a reduced data representation, Strawberry is 10 around times faster than Cufflinks. StringTie uses a flow algorithm for quantification which is very fast compared to the EM algorithm used by Strawberry and Cufflinks. This makes it unlikely for Strawberry to outrun StringTie. Like StringTie and Cufflinks, Strawberry implements the thread-level parallellism which can process several loci at a time to greatly speed up the program.

The lack of gold standard data for the assessment of RNA-Seq applications is still a major problem for the community. The comparisons used in this paper are primarily based on simulated data where we know the ground truth. However, the simulation programs can fall short of resembling real data in various ways, including sequencing bias, read errors, etc. Numerous studies have shown that bias can be caused by local sequences (e.g., hexamer bias) around the reads [26], position of the reads [27], GC content bias [28], etc. Lahens et al. points out the bias in RNA sequencing is highly unpredictable and might be more complicated than the few reasons aforementioned [29]. Interestingly enough, using the bias correction features in Cufflinks does not lead to an increase in performance even in the real data, all probes Pearson’s r 0.672 vs. 0.670 without/with bias correction (-b option). By allowing different subexon bins to have different conditional probabilities, Strawberry model has more flexibility than models assuming uniform distributions of reads along transcripts and thus may be able to account for the bias problem to some extent. However, the bias problem is still a big problem for RNA-Seq and its application. The solution to this will require effort from both the sequencing and bioinformatics communities.

## Materials and methods

### Assembly problem formulation

Strawberry formulates the assembly problem as an optimization problem, trying to find a parsimonious representation of transcripts which best explains the read alignments. Cufflinks is one of the pioneers which formulates the assembly problem as an optimization problem. Thus, we start with a brief review of the Cufflinks assembly algorithm and use it to introduce Strawberry’s assembly algorithm.

The set of all read-pairs at a locus $R=\{r_{1},\ldots r_{m}\}$ forms a partially ordered set in which *r*_*i*_ ≤ *r*_*j*_ if and only if the start position, in the transcription direction, of *r*_*i*_ is less than or equal to *r*_*j*_ and the two are compatible (can arise from the same transcript). In brief, two read-pairs are incompatible if they imply two different introns and the two introns overlap (cannot arise from the same isoform) [1]. Cufflinks defines a read-pair path *p* as a subset of R, an ordered set of read-pairs {*r*_*a*_1__, …, *r*_*a*_*k*__} with *r*_*a* − 1_ ≤ *r*_*a*_ for all 1 < *a* ≤ *k*. Then, the assembly problem is equivalent to finding the read-pair path cover *C* = {*p*_1_, …, *p*_*n*_}, where ‖*C*‖_0_ is minimized and

$$\begin{array}{c} \forall r\in R,\exists p\in C\wedge p\neq \emptyset ,\;\text{such}\;\text{that}\;r\;\text{is}\;\text{in}\;p. \end{array}$$

The final estimated path cover $\hat{C}$ corresponds to the set of assembled transcripts. This is a canonical computer science problem known as the Minimum Path Cover (MPC) problem [30]. Cufflinks uses a maximum matching algorithm in bipartite graphs to solve the MPC problem [1].

Instead of working with individual read-pairs, Strawberry uses a sparse representation called splicing graphs, a common feature of genome-guided methods. Heber et al. defines a splicing graph *G* = (*V*, *E*) as a directed acyclic graph (DAG) on the set of transcribed positions *V* and edge set *E* [31]. *G* contains an edge from *v*_*i*_ to *v*_*j*_ if and only if *v*_*i*_ < *v*_*j*_ and they have consecutive positions in at least one transcript. The graph *G* can be refined by collapsing consecutive vertices if all of them have only one outgoing edge and one ingoing edge. When doing so, the vertices *V* become exons (or subexons) and edges *E* become introns [31]. We use the term, subexons, to refer to such entities throughout this paper to avoid confusion with real biological exons. Note that subexons are ordered such that *v*_*i*_ < *v*_*j*_ if subexon *v*_*i*_ starts upstream of subexon *v*_*j*_. Furthermore, a read-pair path can be mapped to an ordered collection of subexons, which we call a subexon path.

The splicing graph can be constructed from either a set of transcripts or from read-pairs. Under the assembly mode, Strawberry builds splicing graphs from read-pairs and then assembles the nodes (subexons) into transcripts. Under the splicing graph representation, a similar MPC problem arises on the subexon level. Since the splicing graph is a sparse representation of the read-pairs, assembly on the splicing graph is more time efficient than assembly with the read-pairs. This subexon representation also has a positive impact on quantification, since read-pair counts on subexons can be seen as compact sufficient statistics for our quantification model. The idea of quantification is discussed in more detail in the quantification section.

Our flow network algorithm requires some modifications on the splicing graph. A source node *v*_*s*_ connecting to all subexon(s) at the 5’ end site(s), and a target node *v*_*t*_ connecting to the subexon(s) having at the 3’ end site(s) are added to the splicing graph. We use the word (*s*, *t*)-path (in order to reserve the use of subexon path for quantification) to refer to an ordered set of subexons from *v*_*s*_ to *v*_*t*_, inclusive. Notice that *v*_*s*_ and *v*_*t*_ are not real exons. Our new MPC problem on the splicing graph can be defined as finding a minimum set of (*s*, *t*)-paths which can cover every subexon at least once. The purpose of including nodes, *v*_*s*_ and *v*_*t*_, is to remove partial or incomplete transcripts. In other words, each full transcript corresponds to a (*s*, *t*)-path which flows from a promoter region (*v*_*s*_) to a terminator (*v*_*t*_).

### Constructing a weighted splicing graph

**Fig 8:**
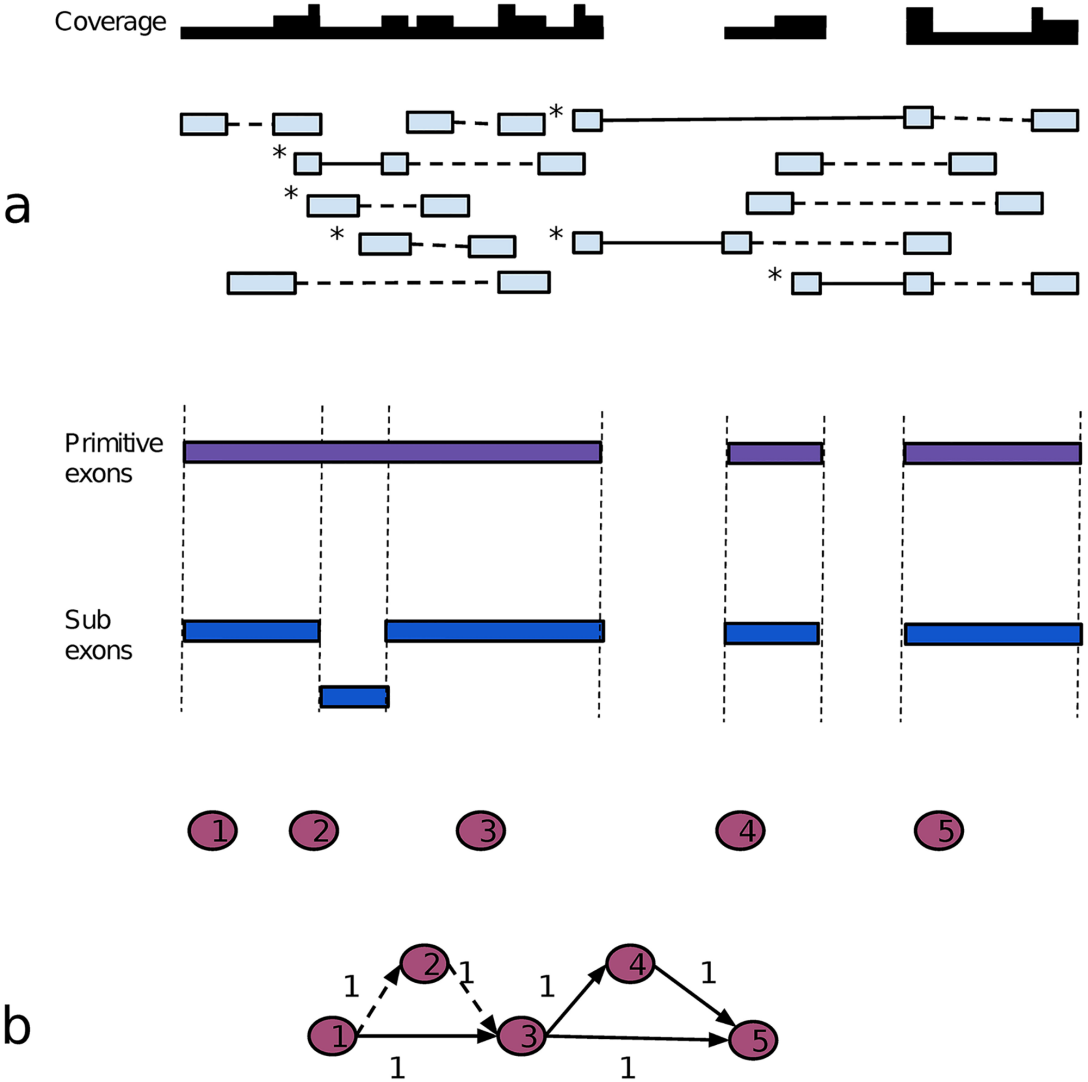
Translation of read alignments into a splicing graph. (a) Eleven imaginary aligned paired-end reads (or read-pairs) are represented by light blue boxes intersected by solid lines, which indicate splicing junctions, and broken lines, which indicates gap sequences. Above the read-pairs, the coverage plot is shown. The white regions have zero coverage. Below the read-pairs, three primitive exons are shown as purple boxes and five subexons in dark blue, numbered from 1–5. (b) The splicing graph constructed from part (a). The numbered nodes in the splicing graph are subexons from part (a). Dashed Arrows represent the non-intron edges and solid arrows indicate the intron edges. The numbers next to edges are the weights(number of read-pairs supports). A read-pair that contributes to an edge weight is stressed using an asterisk near its upper-left corner. All the arrows also indicate the transcription direction. The source node and target node in the splicing graph are not shown.

To define nodes and edges in the splicing graph, Strawberry separately retrieves primitive exons from the coverage data and retrieves introns from junction alignments. A primitive exon is defined as a continuous stretch of genomic positions covered by reads. An intron is defined as a unique junction alignment. The introns are then used to cut the primitive exons into subexons which are the final nodes defined in the splicing graph (Fig 8). However, in simulated data, many inferred introns are not real because of false junction calls by aligners. There is evidence these false calls also appear in real data [32]. Strawberry uses the same criteria to pre-filter introns as in Cufflinks [1]. The thresholds are arbitrary but work well in practice. Putative introns are discarded if any of the following apply.

- More than 70% of the reads supporting an intron are not uniquely aligned.
- If two introns overlap and one’s expression is less than 5% of the other, then the one with lower expression is removed. Intron expression is defined as the total number of junction reads.
- The number of small overhang reads supporting a junction is likely to be low under the assumption that reads are distributed uniformly along their parent transcripts. A small overhang read is a particular junction read where one end of the read is mapped within a small distance (we use 6 bp) of a subexon-intron boundary. The expected number of small overhang reads is calculated from a binomial distribution, Bin(*n*, *p*), where *n* is the total junction reads and $p=\frac{2s}{l-1}$, *s* being the small overhang distance and *l* being read length. When *n* is large (e.g., >100), we use the normal approximation *N*(*np*, *np*(1 − *p*)).

Next, nodes (subexons) are connected in the splicing graph. Each subexon is either fully contained or excluded in any transcript. Two subexons are connected by an edge, which does not necessarily represent an intron, when they are consecutive in their genomic coordinates (see Fig 8). For the non-intron edges, the number of reads covering at least 6 bp of both subexons is used as the edge weight representing the support for these two subexons being in the same transcript. For the intron edges, the weight is simply the total junction read number. In the implementation, Strawberry negates the weight and adds the maximum weight to make all weights positive. The algorithm, described next, will solve for the minimum total weight.

### Optimization with flow network

**Fig 9:**
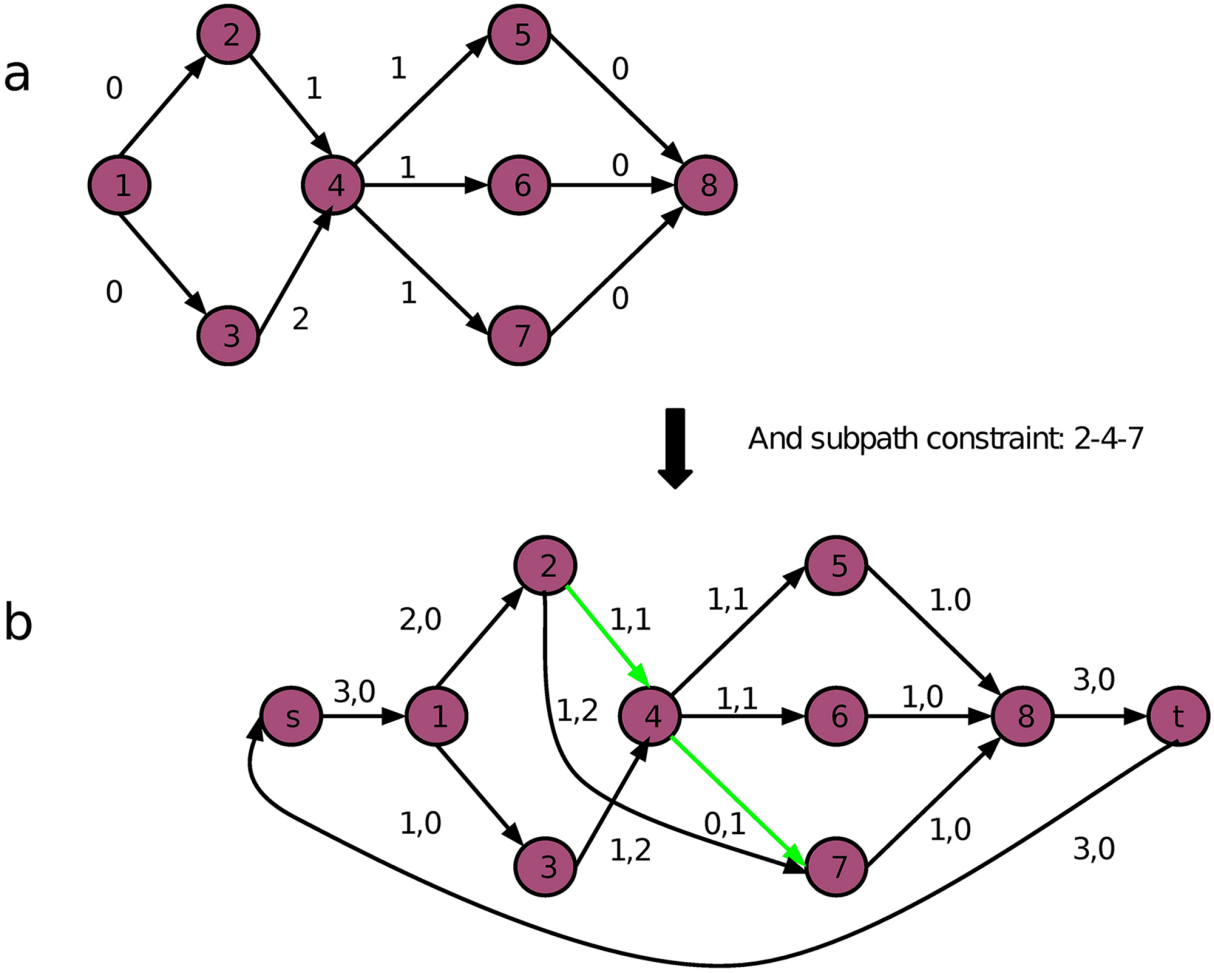
An input flow network with a subpath constraint {2-4-7}. (a), the number next to an edge is the edge cost. For every edge *e*, the edge constraint implies 1 ≤ *f*(*e*) ≤ inf. (b), the transformed min-flow circulation network. The 2-tuple (a, b) next to each edge indicates the optimal flow on the edge and the edge cost respectively. After Step 3, the path constraints set is *P*^sub^ = {(1, 2), (1, 3), (2, 4, 7), (4, 5), (4, 6), (5, 8), (6, 8), (7, 8)}. Two edges no longer in the constraint set are shown in green. For these two edges, the minimum flow requirement is 0; for the rest of edges, it is 1. Two dummy nodes, *s* and *t*, are added to complete the circulation. The number of flows after decomposition is equal to the minimum flow which is 3.

We have reformulated the problem on a splicing graph *G*, where (*s*, *t*)-paths (full-length transcripts) are ordered collections of subexons, and we seek a minimum path cover (MPC) of *G*. The ordinary MPC problem is not a good fit for the splicing graph since it only requires that every node (subexon) is covered at least once, leaving the possibility that some edges (indicating two subexons are consecutive in the transcriptome) might not be covered. Also, a read-pair (due to the unsequenced proportion) can span two non-consecutive nodes. These non-consecutive nodes (if they exist) constitute a subpath (Fig 9), denoted by *p*^*sub*^, that also must be covered by at least one (*s*, *t*)-path in the cover. All the edges and subpaths constitute the constraints in a Constrained MPC (CMPC) problem. An efficient algorithm for solving the Constrained MPC (CMPC) problem has been advanced [33].

**Definition 1** *CMPC problem*. *Given a DAG G with nodes V*(*G*) *and edges E*(*G*), *and a weight w*(*e*) *for each e* ∈ *E*(*G*), *and a set of subpaths* $\left\{p_{j}^{sub}|j\in 1,\ldots ,t\right\}$ *the task is to find a minimum number of k directed paths* {*p*_*i*_|*i* ∈ 1, …, *k*} *in G such that*

- *Every node in V*(*G*) *occurs at least once in some p*_*i*_.
- *Every edge in E*(*G*) *occurs at least once in some p*_*i*_.
- *Every path* $p_{j}^{sub}$ *is entirely contained in some p*_*i*_.
- *Every path p*_*i*_ *starts in v*_*s*_ *and ends in v*_*t*_, *where v*_*s*_ *and v*_*t*_ *are the source and target nodes of G*.
- $\sum_{i=1}^{k}\sum_{e\in p_{i}}w\left(e\right)$ *is minimum among all solutions of k paths*.

Rizzi et al. showed that the CMPC problem can be reduced to the MPC problem with node constraints [33]. The MPC with node constraints can be found using one of the well established flow network algorithms, e.g., the min-cost circulation flow algorithm [30], where a strong polynomial time solution is guaranteed. In a nutshell, a flow network is a DAG *G* = (*V*, *E*) with source *v*_*s*_ ∈ *V*(*G*) and target *v*_*t*_ ∈ *V*(*G*), where every edge *e* ∈ *E*(*G*) has an upper *u*(*e*) and lower *l*(*e*) capacity limit and flow *f*(*e*) associated with it. The solution to a flow network problem is to construct a map, *f*: *E* → *R*, which maps an edge to a real number or an integer, called a flow. The flow decomposition theorem (see, e.g., [30]) guarantees the flow network can be used to solve the MPC problem. It says that the flow *f*(*e*) on edge *e* can be decomposed into a set of flows on the (*s*, *t*)-path. However the decomposition is not unique, which we overcome using a greedy algorithm.

**Algorithm 1** *Constrained Minimum Path Cover Algorithm (CMPC)* [33]

1. *Add edges to the subpath constraints*. *Let* $P^{sub}=\left\{p_{i}^{sub}\right\}$ *denote the set of subpath constraints*. *Grow the P*^*sub*^ *to include all edges as subpath constraints*.
2. *Drop duplicates*. *For every pair of path constraints* $p_{i}^{sub}$ *and* $p_{j}^{sub}$, *set P*^*sub*^ *to* $P^{sub}\setminus p_{i}^{sub}$, *if* $p_{i}^{sub}$ *is contained in* $p_{j}^{sub}$.
3. *For every original path constraint* $p_{i}^{sub}$ *which starts at node u and ends at node v and* (*u*, *v*) ∉ *E*(*G*), *do*:
  - *E*(*G*) ≔ *E*(*G*) ∪ {(*u*, *v*)}. *Add a new edge* (*u*, *v*) *directly from the start node of the subpath to the end node of the subpath*.
  - *Set the lower and upper bounds for this new edge*: *lower*(*u*, *v*) = 1 *and upper*(*u*, *v*) = inf.
  - *The weight of the new edge is the sum of weights of the original subpath*: $w\left(u,v\right)=\sum_{e\in p_{i}^{sub}}w\left(e\right)$.
4. *For each e* ∈ *E*(*G*) *and e* ∉ *P*^*sub*^, *set lower*(*e*) = 0 *and upper*(*e*) = inf.
5. *Add an edge* (*v*_*t*_, *v*_*s*_) *from sink node v*_*t*_ *to start node v*_*s*_ *to complete the circle*. *Set lower and upper bounds for this edge as well*: *lower*(*t*, *s*) = 0 *and upper*(*t*, *s*) = inf.
6. *Compute a min-weight min-flow circulation on this transformed input G with the following properties*.
  - *G is a flow network which satisfies capacity constraints and flow conservation constraints*.
  - *Min flow*: ∑_*e*∈*E*(*G*)_ *f*(*e*) *is minimum*.
  - *Min weight*: ∑_*e*∈*E*(*G*)_ *w*(*e*) *is minimum*.
7. *Finally*, *the integer flow on edge* (*v*_*t*_, *v*_*s*_) *equals to the achieved min-flow*. *We decompose the flow network into this number of paths and each path corresponds to an assembled transcript*.

Fig 9 demonstrates a toy example of this algorithm.

### Quantification with latent class model

Strawberry’s quantification model is based on the generative model proposed in [1]. As in *Salzman et al*. *2011* [16], Strawberry collapses data into sufficient statistics, but to match the assembly, Strawberry collapses the data into subexon paths, defined on the splicing graph. In theory, for a gene with *w* subexons, Strawberry produces 2^*w*^ − 1 equivalent classes independent of the number of isoforms. In contrast, the number of classes in [16] depends on the number of isoforms. Although *Salzman et al*. *2011* achieves greater collapsing, Strawberry has a richer parameterization and is able to account for nonuniform distribution of the reads along a transcript. Either way, the idea of collapsing greatly reduces the number of observations and speeds up the calculation.

To describe the Strawberry model, we start with the definition of subexon path. A read-pair can be reduced to a unique set of ordered subexons, called a subexon path. The map from read-pair space R to subexon path space S is surjective. Strawberry’s data reduction strategy creates an equivalency between the subexon paths S and a partition of fragments F (and hence reads R). It collapses read-pairs based on the set of subexons they cross. Let $S=\{S_{1},S_{2},\ldots ,S_{L}\}$ be the collection of subexon paths. Subexon paths are equivalent to sets of genomic intervals {[*G*_*sx*_, *G*_*sy*_] | ∀*s* ∈ *S*_*l*_}, where *G*_*sx*_ and *G*_*sy*_ are the smallest and largest genomic positions in subexon *s*. Each observable read-pair *r* can be represented as a 4-tuple, (*u*_5′_, *u*_3′_, *d*_5′_, *d*_3′_), where *u* and *d* represent the upstream and downstream reads, 5′ and 3′ their respective ends, both along the transcription direction. Then we can partition (or project) the R onto *S*, so that a read pair *r* is assigned to a subexon path *S*_*l*_ if and only if *r* overlaps with only subexons in *S*_*l*_ and all subexons forming *S*_*l*_ have been hit by this *r*, i.e., *r* ∈ *S*_*l*_ ⇔ cond.1 ∧ cond.2, where cond.1 = ∀*s* ∈ *S*_*l*_, [*G*_*sx*_, *G*_*sy*_] ∩ [*u*_3′_, *u*_5′_] ≠ ∅ ∨ [*G*_*sx*_, *G*_*sy*_] ∩ [*d*_5′_, *d*_3′_] ≠ ∅ and cond.2 = ∀*j* ∈ [*u*_3′_, *u*_5′_] ∪ [*d*_5′_, *d*_3′_], ∃*s* such that *j* ∈ [*G*_*sx*_, *G*_*sy*_]. This definition ensures each *r* is uniquely assigned to a *S*_*l*_. Notice, if a read pair contains an unsequenced portion, such as the insert, the subexon path of the read-pair is an incomplete observation of the unobserved set of subexons. However, when conditioning on the isoform, a subexon path can become a complete observation of the fragment from which the read-pair is generated. Therefore, an subexon path can be included or excluded from an isoform just like the read-pair. For each gene *g*, we derive a binary matrix *C* with *L*_*g*_ rows and *K*_*g*_ columns, where we assume gene *g* has *L*_*g*_ subexon paths and *K*_*g*_ isoforms and *C*_*kl*_ = 1 if isoform k contains subexon path *l*, otherwise 0. If there are total *n*_*g*_ read-pairs observed for gene *g*, we derive our observation {***y***_*i*_}_*n*_*g*__, where each element ***y***_*i*_ identifies the subexon path of the read-pair *i*, i.e., ***y***_*i*_ is an *L*_*g*_-dimensional vector, one of the standard basis vectors of *L*_*g*_-dimensional Euclidean space. In practice, Strawberry only uses the observed subexon path whose number so *L*_*g*_ is smaller than the theoretical number.

Like the assembly, this model handles one locus from a single sample at a time, allowing maximum parallelization. Our generative model for RNA-Seq is as follows. Transcripts from isoform *k* make up a proportion *η*_*k*_ in the sample. Transcripts are randomly fragmented, and long isoforms produce more fragments than short isoforms. Isoform *k* fragments constitute approximately proportion *π*_*k*_ ≈ *l*_*k*_*η*_*k*_ in the sample. Having estimated ${\hat{\pi }}_{k}$ and knowing *l*_*k*_, we can later retrieve *η*_*k*_ [1]. Given the isoform of origin *k*, the fragment is considered as generated from the underlying subexon path as a one-trial multinomial experiment Mult(1, ***θ***_*k*⋅_), where *θ*_*kl*_ is a conditional probability of the fragment generating from subexon path *l*. For a given read set $R={\left\{y_{i}\right\}}_{n}$, the likelihood can be written as

$$\begin{array}{c} L\left(\pi ,\theta \;|\;R\right)=\prod_{i=1}^{n}\sum_{k=1}^{K}\pi_{k}\prod_{l=1}^{L}\theta_{kl}^{y_{il}} \end{array}$$ (3)

Following the line of generative model of sampling transcripts first and then the fragments conditioning on the transcript and accounting for the read-isoform assignment uncertainty using a mixture model. Strawberry simultaneously estimates the class probability ***π*** and the conditional probability ***θ*** under a EM algorithm [34] framework, while other models [1, 6, 16, 35] assume fixed conditional probability when estimating the class probabilities. Strawberry has a richer set of parameters which allow it to account for the non-uniform distribution of reads along transcripts often observed in real data [27, 36]. Jiang et al. also proposed a model that simultaneously estimates the class probabilities and conditional probabilities for robust estimation of isoform expression [37]. However, their model has far more parameters than ours and uses a penalized likelihood. Because they don’t publish their program, the actual performance of their model is unknown.

### Estimation

We use the EM algorithm proposed for basic latent class models [38] and summarize in algorithm 2:

**Algorithm** **2**

- *Initialize*
  $$\begin{array}{c} \pi_{k}=1/K, \end{array}$$
  $$\begin{array}{c} \theta_{kl}=\sum_{t}\frac{q\left(t\right)\cdot n_{klt}}{l_{k}-t+1}, \end{array}$$
  *where we sum over possible fragment length t conditioning on subexon bin l and isoform k*. *Here*, *q*(⋅) *is the empirical fragment distribution and n*_*klt*_ *is number of possible fragments with length t and l*_*k*_ *is the isoform length*.
- *repeat EM steps until convergence*.
  – *E-step*:
    $$\begin{array}{c} {\hat{n_{kl}}}^{m+1}=\frac{n_{l}{\hat{\pi_{k}}}^{m}{\hat{\theta_{kl}}}^{m}}{\sum_{k=1}^{K}{\hat{\pi_{k}}}^{m}{\hat{\theta_{kl}}}^{m}}. \end{array}$$
  – *M-step*:
    $$\begin{array}{c} {\hat{\pi_{k}}}^{m+1}=\frac{\sum_{l=1}^{L}{\hat{n_{lk}}}^{m+1}}{n}, \end{array}$$
    $$\begin{array}{c} {\hat{\theta_{kl}}}^{m+1}=\frac{{\hat{n_{kl}}}^{m+1}}{\sum_{l=1}^{L}{\hat{n_{kl}}}^{m+1}}. \end{array}$$

The parameter ***θ*** is initialized using the concept of *read type* (same as our read-pair concept) and *sample rate* ***α*** in [16]. The probability of observing a read pair *r* is $\sum_{k=1}^{K}\pi_{k}\alpha_{kr}$ where

$$\begin{array}{c} \alpha_{kr}=\{\begin{array}{cc} \frac{q(t_{k})}{l_{k}-t_{k}+1}, & \text{if}\;r\;\text{is}\;\text{compatible}\;\text{to}\;\text{isoform}\;\text{k}.\\ 0, & \text{if}\;r\;\text{is}\;\text{not}\;\text{compatible}\;\text{to}\;\text{isoform}\;\text{k}. \end{array} \end{array}$$

We use *t*_*k*_ to denote the fragment length of a read-pair under the isoform *k*. Note that Salzman et al.’s model assumes reads are generated uniformly when its isoform of origin is known. Strawberry learns an empirical fragment size distribution *q*(⋅) from a place in genome (> 2*kb*) where no alternative splice sites exist according to the read alignments. If the input is single end reads, Strawberry relies on the users to define a Gaussian distribution for the fragment length. We assume the random fragmentation step in sample preparation leads to a nearly Gaussian distribution [36], but it is common to approximate the distribution using an empirical one [1].

Strawberry calculates the initial estimate of *θ*_*kl*_ for each pair of isoform *k* and subexon path *l* by summing *α*_*kr*_ over all potential read-pairs on subexon path *l* including the ones that are not observed:

$$\begin{array}{c} \theta_{kl}=\{\begin{array}{cc} \sum_{r\in S_{l}}\alpha_{kr}, & \text{if}\;C_{kl}=1.\\ 0, & \text{if}\;C_{kl}=0. \end{array} \end{array}$$ (4)

**Fig 10:**
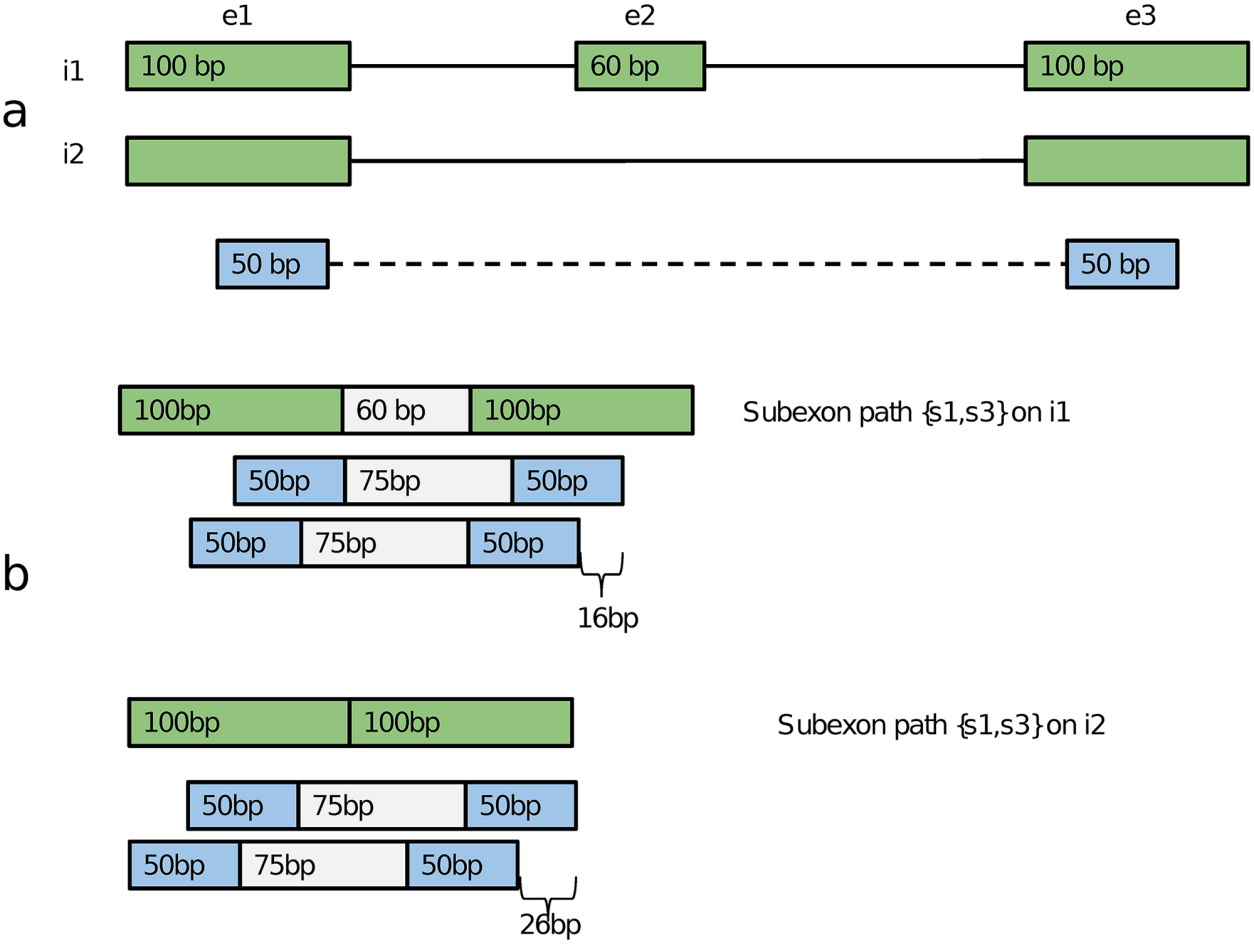
(a), a gene with three subexons and two isoform are shown. The length of i1 is 260 bp, i2 200 bp. A paired-end read (or read-pair) is represented by light blue boxes intersected by broken lines, which indicates gap sequences. The read length is 50x2 bp. (b) A subexon path {*s*_1_, *s*_3_} applies to both isoform. When on i1, this subexon path implies three subexons with the one in middle shown in gray. Consider a fixed size fragment with gap size 75 bp(shown in gray) and total fragment length 175 bp. This particular fragment can arise from 16 different positions from subexon path {*s*_1_, *s*_3_} on i1 and 26 different positions from subexon path {*s*_1_, *s*_3_} on i2.

The summation in Eq 4 requires summing over all possible fragment lengths and conditioning on a fragment length, the possible 5’ end which *r* can be generated from a given subexon path and transcript combination (Fig 10).

### Implementation

Strawberry was written in C++14 and utilizes features such as threading library for parallelization. *Lemon* [39], a C++ graph template library, was used in assembly and *Eigen3* (http://eigen.tuxfamily.org), a C++ template library for linear algebra, was used in quantification. Strawberry is available as a free software at https://github.com/ruolin/strawberry under the MIT license.

### Conclusion

This paper introduced Strawberry, a fast, accurate genome-guide assembler and quantification tool for RNA-Seq data. It facilitates transcriptome assembly and calculation of transcript-level expression. Based on our simulation, Strawberry not only recovers more true transcripts while achieving the same false discovery rate in assembly compared to two other leading methods but also outperforms them in terms of the quantification accuracy. Using the real data from a highly cited method comparison study, we again show that Strawberry beats Cufflinks and StringTie by convincing margins. The other advantage of Strawberry is its speed and good scalability, makes it an intriguing candidate when processing large dataset (e.g., >100 million reads). It takes 12.35 min for Strawberry to process 100 million input RNA-Seq reads while a simple Linux program *wc* takes 8.69 min. Strawberry achieves this level of speed and accuracy through applying the min-cost, min-flow algorithm to assembly, a reduced data representation to subexon path counts which arise naturally from the splicing graph and latent class model used in the quantification step. Strawberry is written in C++14 and is fully self-contained. The installation does not require any pre-installation packages except for *g++ compiler* and *CMake*. Strawberry applies to both single-end and paired-end libraries, and also supports strand-specific protocols.

## Supporting information

S1 Fig RD60 assembly result. Recall and precision at the nucleotide, exon, intron and transcript level for StringTie, Cufflinks and Strawberry at RD60 data.
(TIF)

S2 Fig RD25 assembly result. Recall and precision at the nucleotide, exon, intron and transcript level for StringTie, Cufflinks and Strawberry at RD25 data.
(TIF)

S3 Fig RD60 quantification result. Frequency plot of Proportional correlation, Spearman correlation, Mean Absolute Relative Difference (MARD) for the 10 replicates in RD60 data.
(EPS)

S4 Fig RD25 quantification result. Frequency plot of Proportional correlation, Spearman correlation, Mean Absolute Relative Difference (MARD) for the 10 replicates in RD25 data.
(EPS)

S5 Fig Box plots of F1 scores at the exon and intron level. StringTie, Cufflinks and Strawberry were evaluated on data *GEU*, which is a simulated Human RNA-Seq data set.
(TIF)

S6 Fig Frequency plot of Proportional correlation, Spearman correlation, Mean Absolute Relative Difference (MARD) for the 6 samples in *GEU*, which is a simulated Human data. These comparisons include only the reconstructed transcripts that fully match the known transcripts.
(TIF)

## References

[1] Trapnell C, Williams BA, Pertea G, Mortazavi A, Kwan G, van Baren MJ, et al Transcript assembly and quantification by RNA-Seq reveals unannotated transcripts and isoform switching during cell differentiation. Nat Biotechnol. 2010;28(5):511–515. doi: 10.1038/nbt.1621 20436464 10.1038/nbt.1621 PMC3146043

[2] Pertea M, Pertea GM, Antonescu CM, Chang TC, Mendell JT, Salzberg SL. StringTie enables improved reconstruction of a transcriptome from RNA-seq reads. Nat Biotechnol. 2015;33(3):290–295. doi: 10.1038/nbt.3122 25690850 10.1038/nbt.3122 PMC4643835

[3] Bernard E, Jacob L, Mairal J, Vert JP. Efficient RNA isoform identification and quantification from RNA-Seq data with network flows. Bioinformatics. 2014;30(17):2447–2455. doi: 10.1093/bioinformatics/btu317 24813214 10.1093/bioinformatics/btu317 PMC4147886

[4] Patro R, Duggal G, Love MI, Irizarry RA, Kingsford C. Salmon provides accurate, fast, and bias-aware transcript expression estimates using dual-phase inference. bioRxiv. 2016;

[5] Love MI, Hogenesch JB, Irizarry RA. Modeling of RNA-seq fragment sequence bias reduces systematic errors in transcript abundance estimation. Nat Biotechnol. 2016;34(12):1287–1291. doi: 10.1038/nbt.3682 27669167 10.1038/nbt.3682 PMC5143225

[6] Rossell D, Stephan-Otto Attolini C, Kroiss M, Stocker A. QUANTIFYING ALTERNATIVE SPLICING FROM PAIRED-END RNA-SEQUENCING DATA. Ann Appl Stat. 2014;8(1):309–330. doi: 10.1214/13-AOAS687 24795787 10.1214/13-aoas687 PMC4005600

[7] Roberts A, Pachter L. Streaming fragment assignment for real-time analysis of sequencing experiments. Nat Methods. 2013;10(1):71–73. doi: 10.1038/nmeth.2251 23160280 10.1038/nmeth.2251 PMC3880119

[8] Li B, Dewey CN. RSEM: accurate transcript quantification from RNA-Seq data with or without a reference genome. BMC Bioinformatics. 2011;12(1):323 doi: 10.1186/1471-2105-12-323 21816040 10.1186/1471-2105-12-323 PMC3163565

[9] Bray NL, Pimentel H, Melsted P, Pachter L. Near-optimal probabilistic RNA-seq quantification. Nat Biotechnol. 2016;34(5):525–527. doi: 10.1038/nbt.3519 27043002 10.1038/nbt.3519

[10] Liu R, Loraine AE, Dickerson JA. Comparisons of computational methods for differential alternative splicing detection using RNA-seq in plant systems. BMC Bioinformatics. 2014;15(1):364 doi: 10.1186/s12859-014-0364-4 25511303 10.1186/s12859-014-0364-4 PMC4271460

[11] Tomescu AI, Kuosmanen A, Rizzi R, Makinen V. A novel min-cost flow method for estimating transcript expression with RNA-Seq. BMC Bioinformatics. 2013;14 Suppl 5:S15 23734627 10.1186/1471-2105-14-S5-S15 PMC3622638

[12] Mezlini AM, Smith EJ, Fiume M, Buske O, Savich GL, Shah S, et al iReckon: simultaneous isoform discovery and abundance estimation from RNA-seq data. Genome Res. 2013;23(3):519–529. doi: 10.1101/gr.142232.112 23204306 10.1101/gr.142232.112 PMC3589540

[13] Li W, Feng J, Jiang T. IsoLasso: a LASSO regression approach to RNA-Seq based transcriptome assembly. J Comput Biol. 2011;18(11):1693–1707. doi: 10.1089/cmb.2011.0171 21951053 10.1089/cmb.2011.0171 PMC3216102

[14] Garber M, Grabherr MG, Guttman M, Trapnell C. Computational methods for transcriptome annotation and quantification using RNA-seq. Nat Methods. 2011;8(6):469–477. doi: 10.1038/nmeth.1613 21623353 10.1038/nmeth.1613

[15] Song L, Florea L. CLASS: constrained transcript assembly of RNA-seq reads. BMC Bioinformatics. 2013;14 Suppl 5:S14 doi: 10.1186/1471-2105-14-S5-S14 23734605 10.1186/1471-2105-14-S5-S14 PMC3622639

[16] Salzman J, Jiang H, Wong WH. Statistical Modeling of RNA-Seq Data. Stat Sci. 2011;26(1). doi: 10.1214/10-STS343 24307754 10.1214/10-STS343 PMC3846358

[17] Lamesch P, Berardini TZ, Li D, Swarbreck D, Wilks C, Sasidharan R, et al The Arabidopsis Information Resource (TAIR): improved gene annotation and new tools. Nucleic Acids Res. 2012;40(Database issue):D1202–1210. doi: 10.1093/nar/gkr1090 22140109 10.1093/nar/gkr1090 PMC3245047

[18] Griebel T, Zacher B, Ribeca P, Raineri E, Lacroix V, Guigo R, et al Modelling and simulating generic RNA-Seq experiments with the flux simulator. Nucleic Acids Res. 2012;40(20):10073–10083. doi: 10.1093/nar/gks666 22962361 10.1093/nar/gks666 PMC3488205

[19] Kim D, Pertea G, Trapnell C, Pimentel H, Kelley R, Salzberg SL. TopHat2: accurate alignment of transcriptomes in the presence of insertions, deletions and gene fusions. Genome Biol. 2013;14(4):R36 doi: 10.1186/gb-2013-14-4-r36 23618408 10.1186/gb-2013-14-4-r36 PMC4053844

[20] Kim D, Langmead B, Salzberg SL. HISAT: a fast spliced aligner with low memory requirements. Nat Methods. 2015;12(4):357–360. doi: 10.1038/nmeth.3317 25751142 10.1038/nmeth.3317 PMC4655817

[21] Frazee AC, Jaffe AE, Langmead B, Leek JT. Polyester: simulating RNA-seq datasets with differential transcript expression. Bioinformatics. 2015;31(17):2778–2784. doi: 10.1093/bioinformatics/btv272 25926345 10.1093/bioinformatics/btv272 PMC4635655

[22] Lappalainen T, Sammeth M, Friedlander MR, ’t Hoen PA, Monlong J, Rivas MA, et al Transcriptome and genome sequencing uncovers functional variation in humans. Nature. 2013;501(7468):506–511. doi: 10.1038/nature12531 24037378 10.1038/nature12531 PMC3918453

[23] Burset M, Guigo R. Evaluation of gene structure prediction programs. Genomics. 1996;34(3):353–367. doi: 10.1006/geno.1996.0298 8786136 10.1006/geno.1996.0298

[24] Steijger T, Abril JF, Engstrom PG, Kokocinski F, Hubbard TJ, Guigo R, et al Assessment of transcript reconstruction methods for RNA-seq. Nat Methods. 2013;10(12):1177–1184. doi: 10.1038/nmeth.2714 24185837 10.1038/nmeth.2714 PMC3851240

[25] Dobin A, Davis CA, Schlesinger F, Drenkow J, Zaleski C, Jha S, et al STAR: ultrafast universal RNA-seq aligner. Bioinformatics. 2013;29(1):15–21. doi: 10.1093/bioinformatics/bts635 23104886 10.1093/bioinformatics/bts635 PMC3530905

[26] Hansen KD, Brenner SE, Dudoit S. Biases in Illumina transcriptome sequencing caused by random hexamer priming. Nucleic Acids Res. 2010;38(12):e131 doi: 10.1093/nar/gkq224 20395217 10.1093/nar/gkq224 PMC2896536

[27] Roberts A, Trapnell C, Donaghey J, Rinn JL, Pachter L. Improving RNA-Seq expression estimates by correcting for fragment bias. Genome Biol. 2011;12(3):R22 doi: 10.1186/gb-2011-12-3-r22 21410973 10.1186/gb-2011-12-3-r22 PMC3129672

[28] Benjamini Y, Speed TP. Summarizing and correcting the GC content bias in high-throughput sequencing. Nucleic Acids Res. 2012;40(10):e72 doi: 10.1093/nar/gks001 22323520 10.1093/nar/gks001 PMC3378858

[29] Lahens NF, Kavakli IH, Zhang R, Hayer K, Black MB, Dueck H, et al IVT-seq reveals extreme bias in RNA sequencing. Genome Biol. 2014;15(6):R86 doi: 10.1186/gb-2014-15-6-r86 24981968 10.1186/gb-2014-15-6-r86 PMC4197826

[30] Ahuja RK, Magnanti TL, Orlin JB. Network flows: theory, algorithms, and applications. Upper Saddle River (N. J.): Prentice Hall; 1993 Available from: http://opac.inria.fr/record=b1117472.

[31] Heber S, Alekseyev M, Sze SH, Tang H, Pevzner PA. Splicing graphs and EST assembly problem. Bioinformatics. 2002;18 Suppl 1:S181–188. doi: 10.1093/bioinformatics/18.suppl_1.S181 12169546 10.1093/bioinformatics/18.suppl_1.s181

[32] Engstrom PG, Steijger T, Sipos B, Grant GR, Kahles A, Ratsch G, et al Systematic evaluation of spliced alignment programs for RNA-seq data. Nat Methods. 2013;10(12):1185–1191. doi: 10.1038/nmeth.2722 24185836 10.1038/nmeth.2722 PMC4018468

[33] Rizzi R, Tomescu AI, Makinen V. On the complexity of Minimum Path Cover with Subpath Constraints for multi-assembly. BMC Bioinformatics. 2014;15 Suppl 9:S5 doi: 10.1186/1471-2105-15-S9-S5 25252805 10.1186/1471-2105-15-S9-S5 PMC4168716

[34] Dempster AP, Laird NM, Rubin DB. Maximum likelihood from incomplete data via the EM algorithm. Journal of The Royal Statistical Society, Series B. 1977;39(1):1–38.

[35] Li B, Ruotti V, Stewart RM, Thomson JA, Dewey CN. RNA-Seq gene expression estimation with read mapping uncertainty. Bioinformatics. 2010;26(4):493–500. doi: 10.1093/bioinformatics/btp692 20022975 10.1093/bioinformatics/btp692 PMC2820677

[36] Roberts A. Ambiguous fragment assignment for high-throughput sequencing experiments. EECS Department, University of California, Berkeley; 2013 Available from: http://www.eecs.berkeley.edu/Pubs/TechRpts/2013/EECS-2013-177.html.

[37] Jiang H, Salzman J. A penalized likelihood approach for robust estimation of isoform expression. Statistics and Its Interface. 2015; (4):437–445. doi: 10.4310/SII.2015.v8.n4.a3 27239250 10.4310/SII.2015.v8.n4.a3 PMC4879778

[38] McCutcheon AL. Latent class analysis. 64 Sage; 1987.

[39] Porkoláb Z, Pataki N, Dezső B, Jüttner A, Kovács P. Proceedings of the Second Workshop on Generative Technologies (WGT) 2010 LEMON—an Open Source C++ Graph Template Library. Electronic Notes in Theoretical Computer Science. 2011;264(5):23–45.

