# Supplementary figures and images for: Strawberry: Fast and accurate genome-guided transcript reconstruction and quantification from RNA-Seq
Source: PLoS Comput Biol. 2017 Nov 27;13(11):e1005851. doi: 10.1371/journal.pcbi.1005851 (PMC5720828; doi:10.1371/journal.pcbi.1005851)

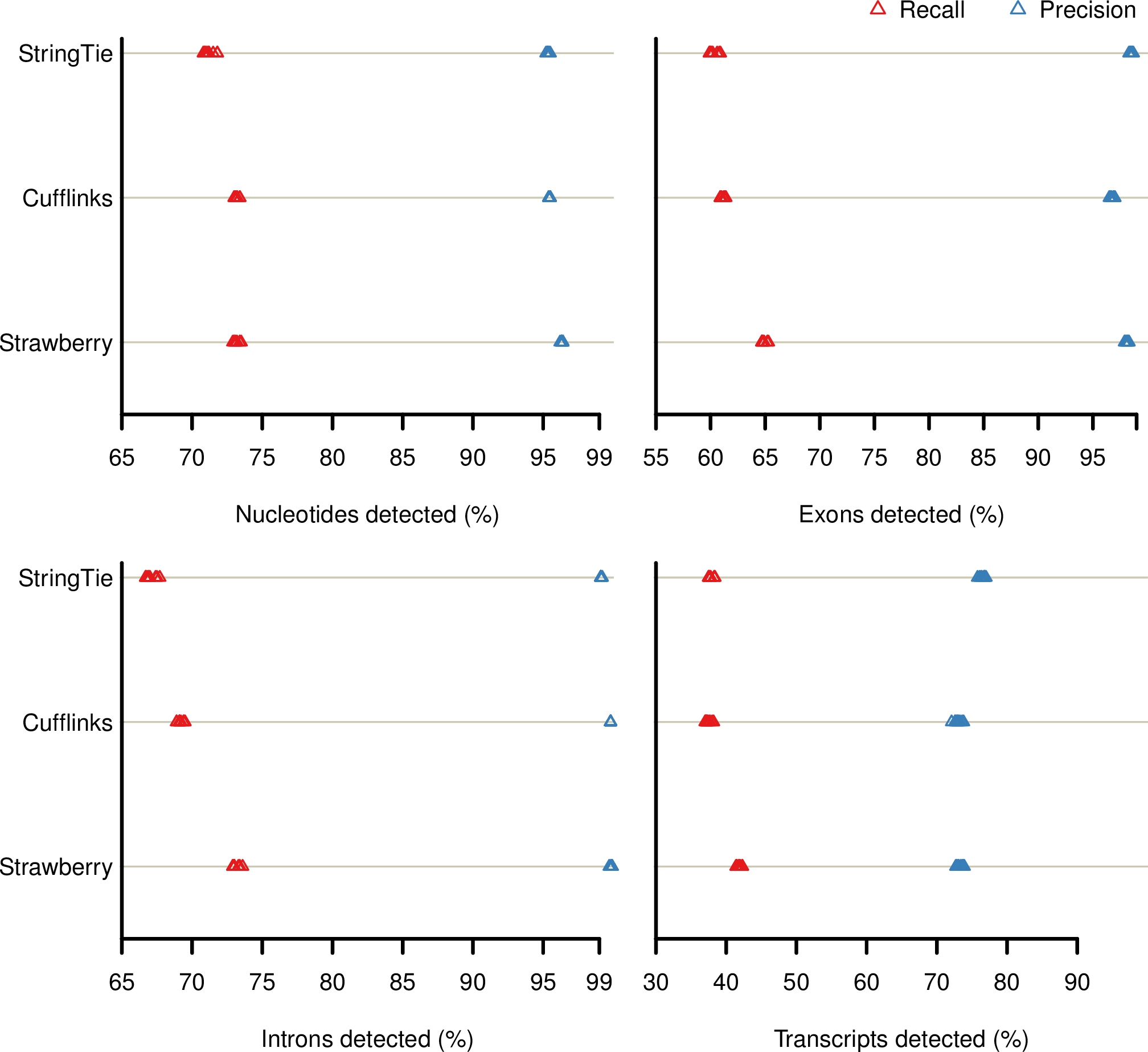

Supplement: S1 Fig — Recall and precision at the nucleotide, exon, intron and transcript level for StringTie, Cufflinks and Strawberry at RD60 data. (TIF) [file pcbi.1005851.s001.tif]

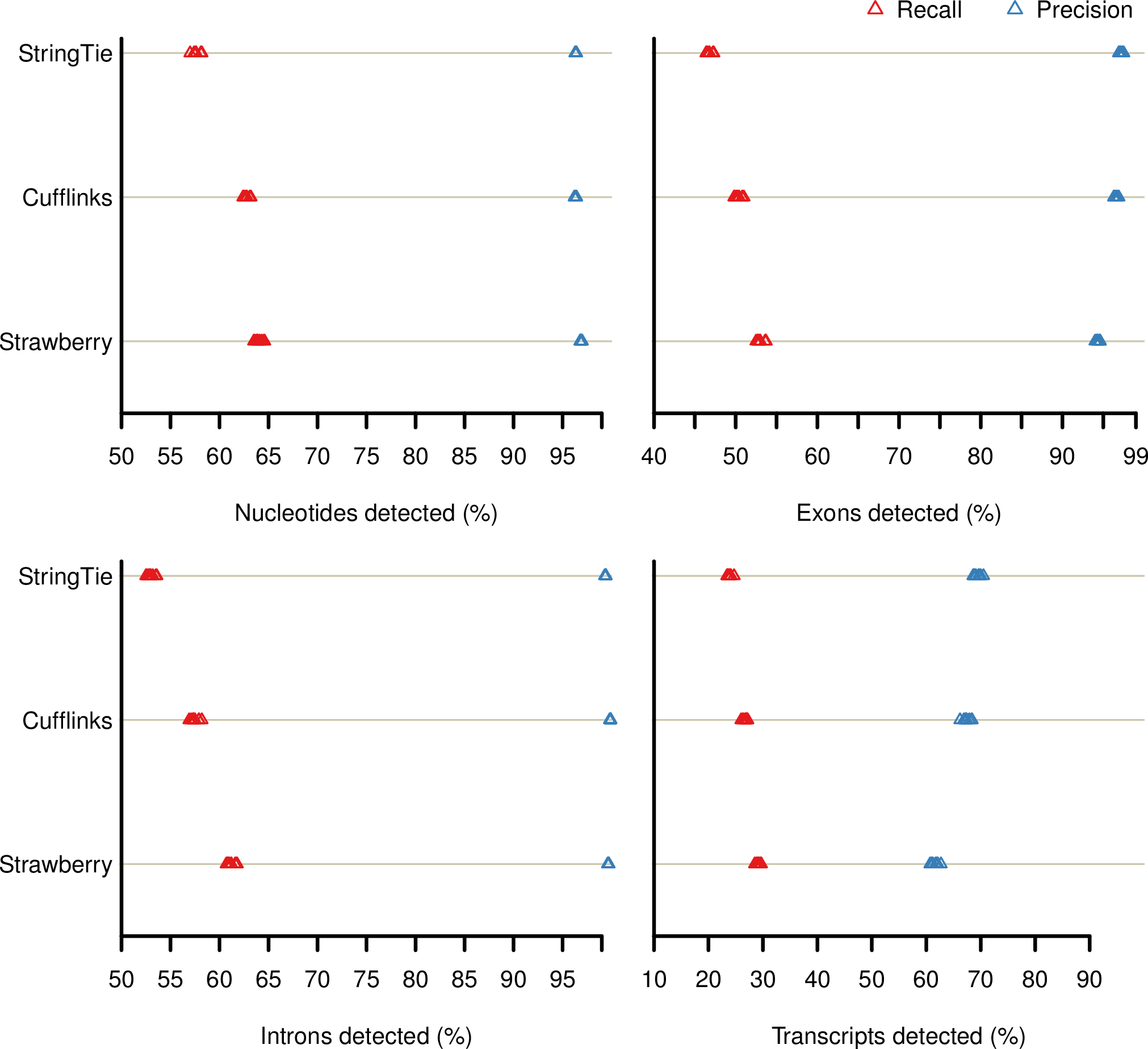

Supplement: S2 Fig — Recall and precision at the nucleotide, exon, intron and transcript level for StringTie, Cufflinks and Strawberry at RD25 data. (TIF) [file pcbi.1005851.s002.tif]

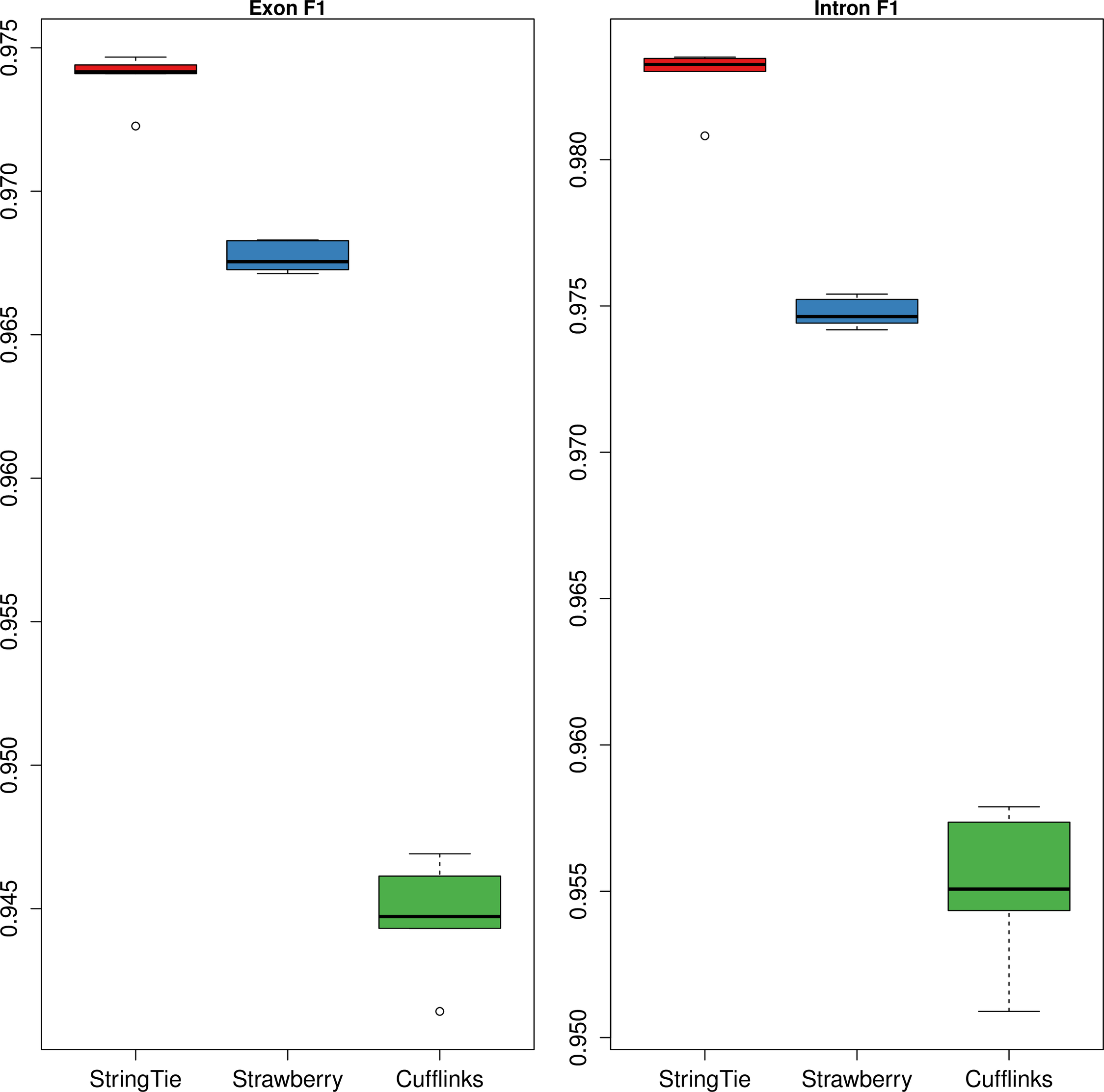

Supplement: S5 Fig — StringTie, Cufflinks and Strawberry were evaluated on data GEU, which is a simulated Human RNA-Seq data set. (TIF) [file pcbi.1005851.s005.tif]

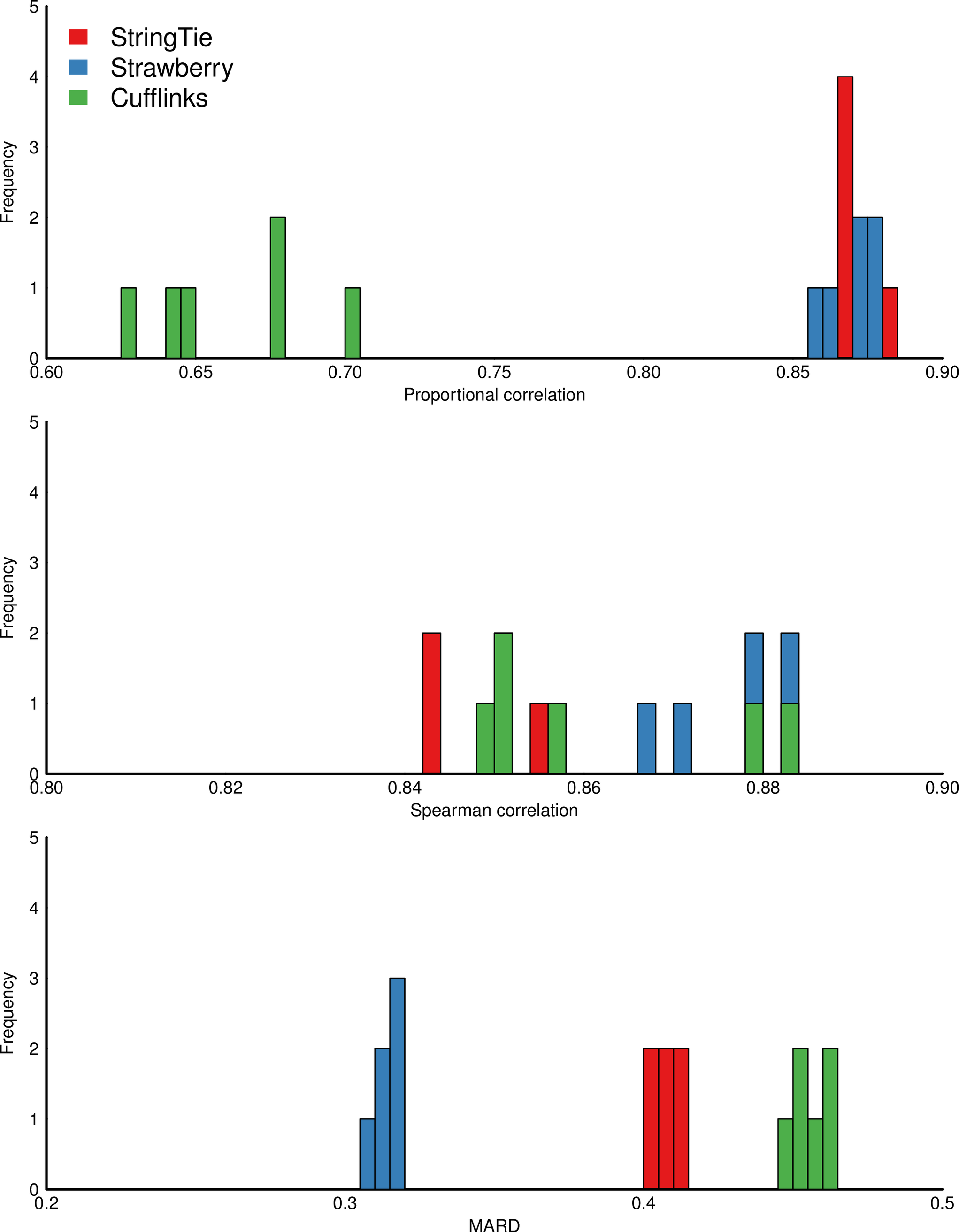

Supplement: S6 Fig — These comparisons include only the reconstructed transcripts that fully match the known transcripts. (TIF) [file pcbi.1005851.s006.tif]
